# Regulated dynamic subcellular GLUT4 localization revealed by proximal proteome mapping in human muscle cells

**DOI:** 10.1242/jcs.261454

**Published:** 2023-12-21

**Authors:** Anuttoma Ray, Jennifer Wen, Lucie Yammine, Jeff Culver, Isabella Supardi Parida, Jeonifer Garren, Liang Xue, Katherine Hales, Qing Xiang, Morris J. Birnbaum, Bei B. Zhang, Mara Monetti, Timothy E. McGraw

**Affiliations:** ^1^ Department of Biochemistry, Weill Cornell Medicine, New York, NY 10021, USA; ^2^ Internal Medicine Research Unit, Pfizer Worldwide Research, Development and Medical, Cambridge, MA 02139, USA; ^3^ Global Biometrics and Data Management, Global Product Development, Pfizer Inc., Cambridge, MA 02139, USA; ^4^ Early Clinical Development Biomedicine AI, Pfizer Worldwide Research, Development and Medical, Cambridge, MA 02139, USA; ^5^ Target Sciences, Pfizer Inc., New York, NY 10016, USA; ^6^ Department of Cardiothoracic Surgery, Weill Cornell Medicine, New York, NY 10021, USA

**Keywords:** GLUT4 trafficking, Human muscle cells, AMPK regulation of GLUT4, GLUT4-proximal proteome

## Abstract

Regulation of glucose transport, which is central for control of whole-body metabolism, is determined by the amount of GLUT4 glucose transporter (also known as SLC2A4) in the plasma membrane (PM) of fat and muscle cells. Physiologic signals [such as activated insulin receptor or AMP-activated protein kinase (AMPK)] increase PM GLUT4. Here, we show that the distribution of GLUT4 between the PM and interior of human muscle cells is dynamically maintained, and that AMPK promotes PM redistribution of GLUT4 by regulating exocytosis and endocytosis. Stimulation of exocytosis by AMPK is mediated by Rab10 and the Rab GTPase-activating protein TBC1D4. APEX2 proximity mapping reveals that GLUT4 traverses both PM-proximal and PM-distal compartments in unstimulated muscle cells, further supporting retention of GLUT4 by a constitutive retrieval mechanism. AMPK-stimulated translocation involves GLUT4 redistribution among the same compartments traversed in unstimulated cells, with a significant recruitment of GLUT4 from the Golgi and trans-Golgi network compartments. Our comprehensive proximal protein mapping provides an integrated, high-density, whole-cell accounting of the localization of GLUT4 at a resolution of ∼20 nm that serves as a structural framework for understanding the molecular mechanisms regulating GLUT4 trafficking downstream of different signaling inputs in a physiologically relevant cell type.

## Teaser

**Summary:** Proximal protein mapping provides a high-resolution, whole-cell account of GLUT4 localization in unstimulated and stimulated human muscle cells, revealing the structural framework for regulation of GLUT4 trafficking.

## INTRODUCTION

Muscle cells and adipocytes are the major sites of postprandial glucose disposal (Klip et al., 2019; Santoro et al., 2021; Sylow et al., 2021). In both cell types, postprandial elevated insulin increases glucose uptake by inducing a redistribution of the GLUT4 glucose transporter (also known as SLC2A4) from intracellular compartments to the plasma membrane (PM) (Klip et al., 2019). GLUT4 redistribution to the PM is impaired in insulin resistance and type 2 diabetes, warranting a thorough understanding of the underlying molecular mechanisms governing GLUT4 translocation in muscle and fat cells. In skeletal muscle, contraction (and exercise) also stimulates glucose uptake, and this too is dependent upon GLUT4 translocation to the PM (Sylow et al., 2021). The molecular and mechanistic overlaps between insulin and contraction signaling inputs to GLUT4 are not well understood. At a functional level it is known that (1) a bout of exercise sensitizes muscle to a subsequent challenge with insulin, and (2) exercise-induced GLUT4 translocation is unaffected by insulin resistance, revealing both similarities and important differences in the control of GLUT4 trafficking downstream of these two physiologically relevant signaling inputs (Richter and Hargreaves, 2013; Bird and Hawley, 2016).

AMP-activated protein kinase (AMPK) is a serine/threonine kinase that acts as a sensor of cellular energy status activated by an increased AMP:ATP concentration ratio (Jeon, 2016). AMPK activation in muscle induces glucose uptake downstream of GLUT4 translocation to the PM (Kjøbsted et al., 2018), and AMPK activation is required for exercise to enhance subsequent muscle insulin sensitivity (Kjøbsted et al., 2017; Sylow et al., 2017); these observations support AMPK as a possible therapeutic target controlling blood glucose (Cokorinos et al., 2017). Despite AMPK and insulin receptor signaling converging on GLUT4 trafficking, the interplay between these signal transduction mechanisms has yet to be described in complete molecular detail.

Two Rab GTPase-activating proteins (GAPs), TBC1D1 and TBC1D4 (also known as AS160), are critical regulators of GLUT4 trafficking through their regulation of Rab proteins (Klip et al., 2019). Rab10 has been shown in studies of cultured adipocytes and adipocyte-specific knockout mice to act downstream of TBC1D4 in insulin-stimulated GLUT4 translocation (Sano et al., 2007; Sadacca et al., 2013; Vazirani et al., 2016; Brumfield et al., 2021), whereas Rab8A has been shown in studies of rodent muscle cells to be required for insulin-stimulated GLUT4 translocation (Sun et al., 2010). The Rab(s) required for AMPK-stimulated GLUT4 translocation in muscle remain to be described, although electrical stimulation-induced GLUT4 translocation in cultured muscle cells requires Rab8A, Rab13 and Rab14 (Li et al., 2018).

Here, we use a human skeletal muscle cell line to investigate the molecular machinery governing GLUT4 trafficking in unstimulated cells and to explore how GLUT4 trafficking is altered by insulin receptor or AMPK activation. We report that insulin induces redistribution of GLUT4 to the PM by accelerating exocytosis, which is consistent with previous studies of rodent muscle cells (Sylow et al., 2021), whereas AMPK stimulation promotes GLUT4 translocation by regulating both exocytosis and endocytosis. Our kinetic studies support a model in which PM GLUT4 is in equilibrium with the PM in all conditions, with the amount of PM GLUT4 determined by the kinetics of GLUT4 trafficking to and from the PM. Because GLUT4 trafficking among distinct membrane compartments controls GLUT4-dependent glucose uptake, understanding this process requires a spatial description of the itineraries of GLUT4 in different physiologic states. Spatial information is usually provided by imaging studies in which colocalizations with established fiduciaries are used. However, imaging studies provide a ‘low-density’ map of GLUT4 localization because no more than a handful of fiduciaries can be used at any one time. Here, we report on the high-density, high-resolution GLUT4-proximal proteome of human skeletal muscle cells generated by APEX2 biotinylation mapping. The GLUT4-proximal protein atlas reveals the intracellular compartments traversed by GLUT4 in unstimulated cells and how the distribution of GLUT4 among those compartments is altered by AMPK activation. These findings provide a framework for studies to define the molecular mechanisms regulating GLUT4 trafficking downstream of distinct signaling inputs relevant to metabolic disease states in a physiologically relevant cell type. The new insights on the regulation and kinetics of GLUT4 translocation leading to increased glucose uptake in response to key physiological stimulations should facilitate the discovery of modulators with therapeutic potential.

## RESULTS

### GLUT4 behavior in cultured human muscle cells

**Fig. 1.**
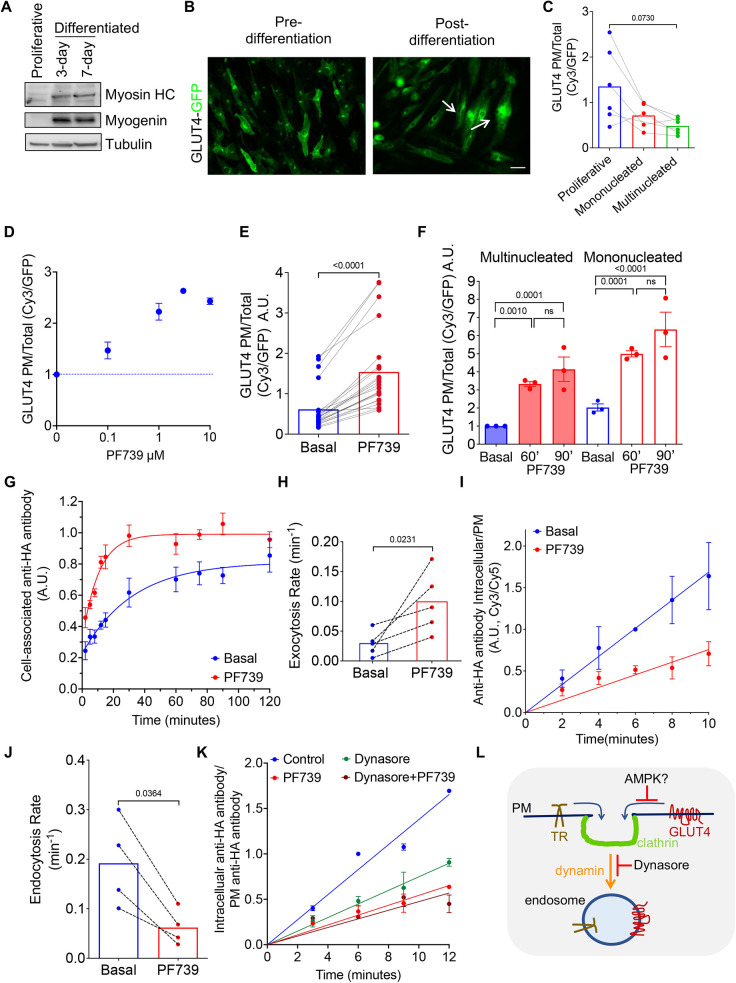
**AMPK regulation of GLUT4 in differentiated human muscle cells.** (A) Western blot of markers of differentiation in SKM-GLUT4 cells at the indicated stages. Tubulin is shown as a loading control. Blots shown are representative of three experiments. HC, heavy chain. (B) Fluorescence microscopy imaging of GFP fluorescence of pre- and post-differentiation (day 3) SKM cells stably expressing HA–GLUT4–GFP by lentiviral transduction. Arrows indicate multinucleated cells. Scale bar: 50 µm. Images shown are representative of three experiments. (C) PM expression of HA–GLUT4–GFP in the indicated classes of SKM-GLUT4 cells. Each data point is the mean value calculated for of at least 40 cells per condition per experiment (*n*=6). The lines link the data for the three conditions from the individual experiments. Bars indicate means. *P*-value calculated using a one-way ANOVA with unequal variances. (D) PF739 dose effect (60 min treatment) on PM expression of HA–GLUT4–GFP in differentiated, multinucleated SKM-GLUT4 cells. Each data point is the mean±s.d. value calculated for of at least 40 cells per condition per experiment. *n*=2, normalized to the basal condition in each experiment. (E) PM expression of HA–GLUT4–GFP in day 3-differentiated, multinucleated SKM-GLUT4 cells treated with 3 µM PF739 for 60 min. Each data point is the PM to total GLUT4 ratio for either PF739-stimulated or unstimulated conditions, calculated from the mean values of at least 40 cells per condition in each of 23 independent experiments. Bars show mean of each condition. *P*-value calculated using a Wilcoxon matched-pairs signed rank test. (F) PM expression of HA–GLUT4–GFP in day 3-differentiated, multinucleated and mononucleated SKM-GLUT4 cells treated with or without 3 µM PF739 for 60 or 90 min (′), as indicated, normalized to the basal condition for multinucleated cells. Basal condition is unstimulated cells. Each data point is the mean value calculated for at least 40 cells per condition per experiment (*n*=3), with the grand mean±s.e.m. shown. One-way ANOVA, *P*<0.0001; pairwise *P*-values in the figure have undergone FDR multiple comparison adjustment (ns, not significant). (G) Exocytosis assay for HA–GLUT4–GFP in day 3-differentiated SKM-GLUT4 cells treated with or without 3 µM PF739 for 60 min. Data are cell-associated anti-HA as a function of incubation time in medium, normalized to the average of 60, 75 and 90 min of basal (unstimulated) condition in each experiment. Data points are means±s.e.m. calculated for at least 40 cells per condition per experiment. *n*=5. Lines show fit to a single exponetial rise to a plateau. (H) Exocytosis rate constants determined from the assays shown in G. Dashed lines join individual experiments. Bar graphs show the mean. *P*-value calculated using a paired, two-tailed Student's two-sample *t*-test. (I) Endocytosis assay for HA–GLUT4–GFP in day 3-differentiated SKM-GLUT4 cells treated with or without 3 µM PF739 for 60 min. Data are a ratio of internal anti-HA (internalized pulse of GLUT4) ratioed to PM GLUT4. Data are normalized to the 6 min time point of the basal (unstimulated) condition in each experiment. Mean±s.e.m., *n*=4. Lines show the fit to a straight line. (J) Endocytosis rate constants determined from the assays shown in I. Dashed lines join individual experiments. Bar graphs show the mean. *P*-value calculated using a paired, two-tailed Student's two-sample *t*-test. (K) Endocytosis assay for HA–GLUT4–GFP in day 3-differentiated SKM-GLUT4 cells in basal (unstimulated) conditions, treated with 3 µM PF739 for 60 min, pre-treated with 100 µM Dynasore for 60 min, or both PF739 and Dyansore for 60 min. PF739 and Dyansore were maintained in the medium during the uptake measurements. Data are a ratio of internal GLUT4 to surface GLUT4, normalized to the 6 min time point of the basal condition in each experiment. Mean±s.e.m., *n*=4. Lines show the fit to a straight line. (L) Cartoon diagram showing endocytosis. See text for details. A.U., arbitrary units.

For our studies of GLUT4, we used human SKM cells, a previously characterized large T antigen-transformed myocyte cell line that can be differentiated *in vitro* into myoblasts and myotube-like cells (Cokorinos et al., 2017). To quantitatively assess GLUT4 behavior, we used a GLUT4 construct engineered with an HA epitope in the first exofacial loop and GFP fused to its cytoplasmic carboxyl domain (HA–GLUT4–GFP) (Dawson et al., 2001; Lampson et al., 2001). HA–GLUT4–GFP in the PM of individual cells was determined by anti-HA immunofluorescence (Cy3 fluorescence) of fixed intact cells, and the total amount of HA–GLUT4–GFP expressed per cell was determined by assaying the GFP fluorescence. Ratiometric analysis of Cy3 and GFP fluorescence for individual cells thus reveals the fraction of total HA–GLUT4–GFP in the PM (Lampson et al., 2001; Karylowski et al., 2004; Blot and McGraw, 2008a,b). SKM cells were transduced with a lentivirus harboring HA–GLUT4–GFP cDNA to obtain a pooled population of cells stably expressing HA–GLUT4–GFP, hereafter referred to as SKM-GLUT4 cells. *In vitro* differentiated SKM-GLUT4 cells expressed myogenin and myosin heavy chain [antibody M4276 stains fast twitch (type II) skeletal muscle cells], two established muscle cell differentiation markers (Miano et al., 1994; Venuti et al., 1995). Incubation of cells in differentiation medium induced expression of these proteins within 3 days, and expression of these markers was maintained for at least 7 days post-differentiation (Fig. 1A). The majority of SKM-GLUT4 cells formed multinucleated cells by day 3 of differentiation, although some cells remained mononucleated despite exposure to the differentiation protocol (Fig. 1B). In both mononucleated and multinucleated cells, HA–GLUT4–GFP was observed in the perinuclear region containing the Golgi and trans-Golgi network (Golgi/TGN), and in puncta (vesicles) dispersed throughout the cytosol. Unless noted otherwise, when we refer to the behavior of GLUT4 in the following discussions of GLUT4 kinetic studies, we are referring to HA–GLUT4–GFP. Because microscopy-based single-cell methods were used to analyze the behavior of GLUT4, multinucleated and mononucleated cells could be analyzed independently. The fraction of GLUT4 at the PM was reduced in differentiated cells compared to that in proliferative myocyte-like SKM-GLUT4 cells, with intracellular accumulation of HA–GLUT4–GFP being the greatest in multinucleated cells (Fig. 1C). In multinucleated cells, 15.5±2.0% (mean±s.e.m.) of total GLUT4 was on the PM. Intracellular retention of GLUT4 is a hallmark of differentiated muscle cells and adipocytes.

### AMPK activation promotes GLUT4 translocation to the PM of differentiated SKM-GLUT4 cells

Treatment of SKM-GLUT4 cells with PF739, an AMPK activator that is non-selective for isoform composition (Cokorinos et al., 2017), stimulated phosphorylation of acetyl-CoA carboxylase (ACC), a substrate of activated AMPK, to the same degree as treatment with 5-aminoimidazole-4-carboxamide ribonucleoside (AICAR), a well-studied AMPK activator (Fig. S1A). In a dose-dependent manner, a 60 min PF739 stimulation of SKM-GLUT4 cells promoted an increase of GLUT4 in the PM (Fig. 1D). A 2.9±0.17-fold (mean±s.e.m.) increase of PM GLUT4 in multinucleated SKM-GLUT4 cells, relative to the level of basal PM GLUT4, was achieved at 3 µM PF739 (Fig. 1E). Both mono- and multi-nucleated SKM-GLUT4 cells responded to PF739 stimulation by translocating GLUT4 to the PM (Fig. 1F). Incubation of cells with PF739 for 90 min did not induce a greater increase in PM GLUT4, demonstrating that a new steady state was reached within 60 min of AMPK stimulation (Fig. 1F). A plot of PM Cy3 versus GFP fluorescence per cell demonstrated that the amount of HA–GLUT4–GFP expressed per cell did not saturate the GLUT4 sorting or trafficking machinery in SKM-GLUT4 cells (Fig. S1B). All kinetic results reported hereafter are from analyses of multinucleated SKM-GLUT4 cells stimulated with 3 µM PF739 for 60 min.

### AMPK activation affects both GLUT4 exocytosis and endocytosis

To explore the mechanism underlying the change in PM GLUT4 following AMPK activation, we characterized GLUT4 exocytosis and endocytosis. Exocytosis can be measured in cells expressing HA–GLUT4–GFP by incubating living cells with an anti-HA antibody and monitoring cell-associated anti-HA antibody as a function of time (Karylowski et al., 2004; Eguez et al., 2005; Martin et al., 2006; Blot and McGraw, 2008a,b). With increasing incubation times, cell-associated anti-HA antibody increases as unbound HA–GLUT4–GFP inside the cell traffics to the PM, where it is bound by antibody. Cell-associated anti-HA antibody accumulation plateaus when all HA–GLUT4–GFP that is in equilibrium between intracellular compartments and the PM has been bound by the antibody (that is, has trafficked once to the PM). Thus, the plateau reflects the size of the GLUT4 pool that is in equilibrium with the PM, and the rate of rise to the plateau is how fast intracellular GLUT4 traffics to the PM (that is, the exocytosis rate constant).

In unstimulated SKM-GLUT4 cells, intracellular GLUT4 was constitutively exocytosed and endocytosed (Fig. 1G–J). Thus, intracellular sequestration of GLUT4 in human SKM cells is a dynamic process determined by the kinetics of internalization and recycling. PF739-mediated activation of AMPK stimulated an acceleration of GLUT4 exocytosis (Fig. 1G). The GLUT4 exocytosis rate constant was increased by ∼3-fold in PF739-stimulated cells (Fig. 1H). In unstimulated SKM-GLUT4 cells, cell-associated anti-HA antibody plateaued at 87.7±1.1% (mean±s.e.m., *n*=5) of the plateau in PF739-stimulated cells, indicating that the effect of AMPK on GLUT4 exocytosis is predominantly to accelerate the movement of GLUT4 that is in equilibrium with the PM of unstimulated cells, although the small difference between the plateaus supports AMPK-dependent mobilization of a pool of GLUT4, constituting ∼12% of the cycling pool in stimulated cells, that is not in equilibrium with the PM in unstimulated cells.

To measure GLUT4 internalization, SKM-GLUT4 cells were pulse labeled with anti-HA antibody. Plotting the ratio of the amount of internal anti-HA antibody to the amount of anti-HA antibody bound to the PM over time yields a straight line with a slope proportional to the internalization rate constant of HA–GLUT4–GFP (Blot and McGraw, 2006; Blot and McGraw, 2008a,b).

PF739-mediated activation of AMPK inhibited GLUT4 internalization by about a third (Fig. 1I,J). Thus, AMPK controls the amount of GLUT4 at the PM by targeting both exocytosis and endocytosis. In rodent muscle cells, GLUT4 is internalized by a dynamin-dependent mechanism (Antonescu et al., 2008). We used Dynasore, a small-molecule inhibitor of dynamin (Kirchhausen et al., 2008), to investigate GLUT4 internalization in SKM-GLUT4 cells. Dynasore inhibition of dynamin-mediated endocytosis in SKM-GLUT4 cells was confirmed by inhibition of the internalization of transferrin receptor (TR, also known as TFRC), a client of dynamin-dependent endocytosis (Fig. S1C). Acute inhibition of dynamin-dependent endocytosis by Dynasore inhibited GLUT4 endocytosis in SKM-GLUT4 cells, which is consistent with a role for dynamin in GLUT4 internalization (Fig. 1K). The effect of Dynasore on GLUT4 internalization was not additive to AMPK activation, providing evidence that AMPK targets dynamin-dependent internalization of GLUT4 (Fig. 1K). However, AMPK inhibition of GLUT4 internalization is not due to a general inhibition of dynamin-dependent endocytosis, because the internalization of TR was unaffected by PF739-mediated activation of AMPK (Fig. S1D). These data support a model in which AMPK regulates GLUT4 internalization at a step upstream of dynamin function in GLUT4 internalization, perhaps targeting GLUT4 clustering in clathrin-coated pits prior to endocytosis (Fig. 1L).

### Insulin stimulation promotes GLUT4 translocation to the PM of differentiated SKM-GLUT4 cells

Insulin control of GLUT4 trafficking has been extensively characterized in rodent muscle cell lines, where it has been shown to induce a near 2-fold increase of PM GLUT4 by stimulating exocytosis (Sylow et al., 2021). Insulin (10 nM) stimulation of differentiated SKM-GLUT4 cells induced phosphorylation of the serine/threonine kinase AKT (here referring to AKT1, AKT2 and AKT3 collectively) and its downstream target TBC1D4 (Fig. S1E–G). TBC1D4 phosphorylation by AKT is required for GLUT4 translocation in adipocytes and muscle cells (Zeigerer et al., 2004; Eguez et al., 2005; Thong et al., 2007; Cartee, 2015). In multinucleated SKM-GLUT4 cells, insulin stimulation induced a 51±17% (mean±s.e.m.) increase of PM GLUT4 relative to that in unstimulated cells, whereas the effect of insulin on PM GLUT4 in mononucleated SKM cells was less (29±14% increase) (Fig. S1H). Insulin-stimulated redistribution of GLUT4 to the PM was accounted for by an increase in the exocytosis rate constant of 37±11% (mean±s.d.), whereas the GLUT4 internalization rate was unchanged (Fig. S1I,J).

### A TBC1D4–Rab10 signaling module links AMPK to regulation of GLUT4 exocytosis in SKM-GLUT4 cells

**Fig. 2.**
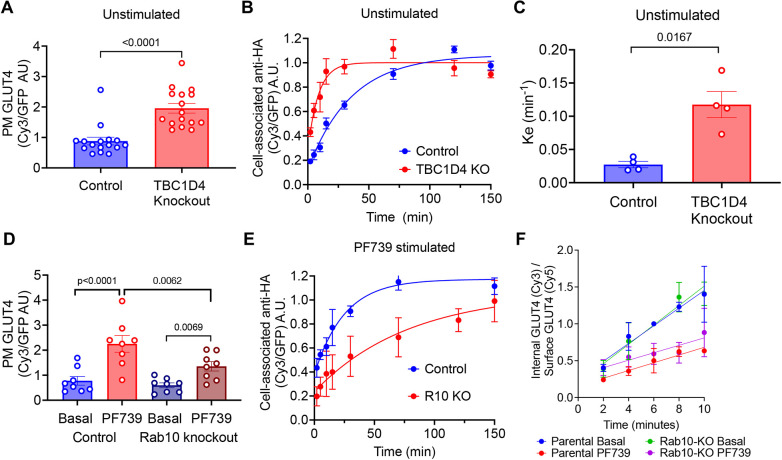
**TBC1D4 and Rab10 are required for AMPK regulation of GLUT4.** (A) PM HA–GLUT4–GFP in day 3-differentiated SKM-CRISPR-Cas9 parental and TBC1D4 knockout (KO) cells stably expressing HA–GLUT4–GFP in basal (unstimulated) conditions. Each symbol is the mean calculated from at least 40 cells in individual experiments (*n*=16). Data are presented as mean±s.e.m. *P*-value calculated using a paired, two-tailed Student's two-sample *t*-test on log-transformed data*.* (B) Exocytosis assay for HA–GLUT4–GFP under basal (unstimulated) conditions in day 3-differentiated SKM-CRISPR-Cas9 parental and TBC1D4 KO cells stably expressing HA–GLUT4–GFP. Data are cell-associated anti-HA as a function of incubation time in medium containing anti-HA antibody, normalized to the average of the 70, 120 and 150 min of basal condition in each experiment. Data are mean±s.e.m., *n*=4. Lines show the fit to a single exponential rise to a plateau. (C) Exocytosis rate constants (*K*_e_), expressed as mean±s.e.m., determined from assays shown in B. *n*=4. *P*-value calculated using a two-tailed Welch's two-sample *t*-test. (D) PM HA–GLUT4–GFP in day 3-differentiated SKM-CRISPR-Cas9 parental cells and Rab10 KO cells stably expressing HA–GLUT4–GFP, treated with or without 3 µM PF739 for 60 min. Each symbol is the mean value of at least 40 cells per experiment (*n*=8). Data are presented as mean±s.e.m., normalized to the basal condition of parental cells. *P*-value two-way ANOVA with unequal variances; *P*<0.0001; pairwise *P*-values in the figure have undergone FDR multiple comparison adjustment. (E) Exocytosis assay for HA–GLUT4–GFP under PF739-stimulated conditions in day 3-differentiated SKM parental and Rab10 (R10) KO cells stably expressing HA–GLUT4–GFP. Data are cell-associated anti-HA as a function of incubation time in medium containing anti-HA antibody, normalized to the average of 70, 120 and 150 min of control condition in each experiment. Data are mean±s.e.m., *n*=4. Lines show the fit to a single exponential rise to a plateau. (F) Endocytosis assay for HA–GLUT4–GFP in day 3-differentiated SKM-CRISPR-Cas9 parental cells and Rab10 KO cells stably expressing GLUT4 treated with or without 3 µM PF739 for 60 min. Data are a ratio of internal anti-HA (internalized pulse of GLUT4) to PM GLUT4. Data are normalized to the 6 min time point of basal (unstimulated) condition in each experiment. Mean±s.e.m., *n*=3. Lines show the fit to a straight line. A.U., arbitrary units.

Our results reveal fundamental differences between responses to insulin and AMPK in the control of GLUT4 in a model human muscle cell line, both in the magnitude of response and mechanisms underlying translocation. The Rab GAP TBC1D4 is a key component of the machinery required for intracellular retention of GLUT4 in rodent adipocytes and muscle cells (Zeigerer et al., 2004; Eguez et al., 2005; Lansey et al., 2012; Dokas et al., 2013; Espelage et al., 2020). In humans, naturally occurring variants and mutations in TBC1D4 are associated with metabolic disruptions, reinforcing its role in regulating GLUT4 traffic to the PM (Dash et al., 2009; Moltke et al., 2014). CRISPR-Cas9-mediated knockout of TBC1D4 in SKM-GLUT4 proliferative cells did not affect differentiation, as assessed by formation of multinucleated cells (Fig. S2A,B). There was a significant increase in the level of PM GLUT4 in basal TBC1D4-knockout cells compared to that in basal control cells (Fig. 2A). This increase was due to an ∼3-fold acceleration of GLUT4 exocytosis in unstimulated TBC1D4-knockout SKM-GLUT4 cells (Fig. 2B,C). These data establish that TBC1D4 functions in human SKM cells to reduce PM GLUT4 in unstimulated conditions by inhibiting GLUT4 exocytosis, consistent with the results of previous studies in adipocytes and rodent muscle cell lines.

In unstimulated cells, TBC1D4 is an active GAP, maintaining its target Rabs in the inactive GDP-bound form. TBC1D4 is phosphorylated by both AKT and AMPK (Treebak et al., 2014; Espelage et al., 2020). Phosphorylation of TBC1D4 inactivates its GAP function, increasing the amount of its target Rabs present in the GTP-bound form. The targets of TBC1D4 GAP activity are Rab2A, Rab8A, Rab10 and Rab14 (Miinea et al., 2005). Knockdown of Rab10 in cultured mouse adipocytes and knockout of Rab10 in primary adipocytes (white and brown) reduce insulin-stimulated GLUT4 translocation, identifying Rab10 as a TBC1D4 target required for GLUT4 translocation in adipocytes (Sano et al., 2007; Vazirani et al., 2016; Picatoste et al., 2021). TBC1D4 is also an AMPK target; therefore, we investigated whether Rab10 has a role in AMPK-induced translocation of GLUT4 in SKM-GLUT4 cells. Knockout of Rab10 did not affect differentiation of SKM-GLUT4 cells, as assessed by formation of multinuclear cells (Fig. S2C,D). PF739-stimulated translocation of GLUT4 to the PM was blunted in Rab10-knockout SKM-GLUT4 cells (Fig. 2D). The reduced translocation resulted from reduced PF739 stimulation of GLUT4 exocytosis in Rab10-knockout SKM-GLUT4 cells (Fig. 2E; Fig. S2E). Rab10 knockout did not affect AMPK inhibition of GLUT4 internalization (Fig. 2F).

It has been previously shown in rat muscle cells that Rab8A, rather than Rab10, is the target of TBC1D4 required for insulin-stimulated GLUT4 translocation (Ishikura and Klip, 2008; Sun et al., 2010). Transient knockdown of Rab8A in differentiated SKM-GLUT4 cells did not affect PF739 stimulated GLUT4 translocation to the PM (Fig. S2F,G), whereas transient Rab10 knockdown blunted PF739-stimulated GLUT4 translocation, as expected based on the Rab10-knockout data (Fig. S2H,I). Hence, as is the case for insulin stimulation of GLUT4 exocytosis in adipocytes, Rab10 is required for AMPK stimulation of GLUT4 exocytosis in human SKM cells. We were unable to interrogate the roles of Rab10 and Rab8A in insulin-stimulated GLUT4 translocation in SKM-GLUT4 cells due to the smaller and more variable net effect of insulin on GLUT4 translocation, as compared to the effect of PF739.

### The GLUT4-proximal proteome in unstimulated SKM cells

Having established that GLUT4 is dynamically retained intracellularly in unstimulated SKM-GLUT4 cells, we next used APEX2 proximity mapping to further explore the trafficking itinerary of GLUT4 in molecular detail. APEX2 is an engineered peroxidase whose substrate is biotin–phenol (Lam et al., 2015). APEX2-generated biotin–phenyl radicals react with electron-dense amino acids to covalently derivatize proteins with biotin. Because the biotin–phenyl radical is highly unstable in aqueous solution, the reaction is limited to ∼20 nm from the site of radical formation. Therefore, proteins within a radius of ∼20 nm of APEX2 tagged to GLUT4 will be biotinylated, and mass spectrometry of streptavidin-isolated biotinylated proteins can be used to identify those proteins. A GLUT4 construct in which the APEX2 peroxidase was fused to the cytosolic carboxyl terminus of GLUT4, replacing GFP of the HA–GLUT4–GFP construct (HA–GLUT4–APEX2), was stably expressed in SKM cells by lentiviral transduction (Fig. S3A). HA–GLUT4–APEX2 stably expressed in SKM cells was localized to the perinuclear region of SKM cells and redistributed to the PM following stimulation with 3 µM PF739 for 90 min (Fig. S3B,C).

To catalog the GLUT4-proximal proteome, SKM cells expressing HA–GLUT4–APEX2 were pre-incubated with APEX2 substrate biotin–phenol for 60 min, followed by incubation with H_2_O_2_ for 60 s. GLUT4-proximal proteins were identified by mass spectrometry analysis of proteins enriched by streptavidin isolation. The GLUT4-proximal proteome was determined by comparing streptavidin-isolated proteins from cells incubated with biotin–phenol and treated with H_2_O_2_ (complete reaction) to those isolated from cells treated with H_2_O_2_ without biotin–phenol pre-incubation (no APEX2 substrate negative control). Analyses of the mass spectrometry intensities from six independent experiments yielded 700 proteins with increased intensity in the biotin–phenol condition relative to the negative control [log_2_(intensity full reaction/intensity no biotin–phenol)>0] and a Benjamini–Hochberg false discovery-corrected *P*-value of less than 0.05 (*P*_adj_<0.05) (Table S1).

**Fig. 3.**
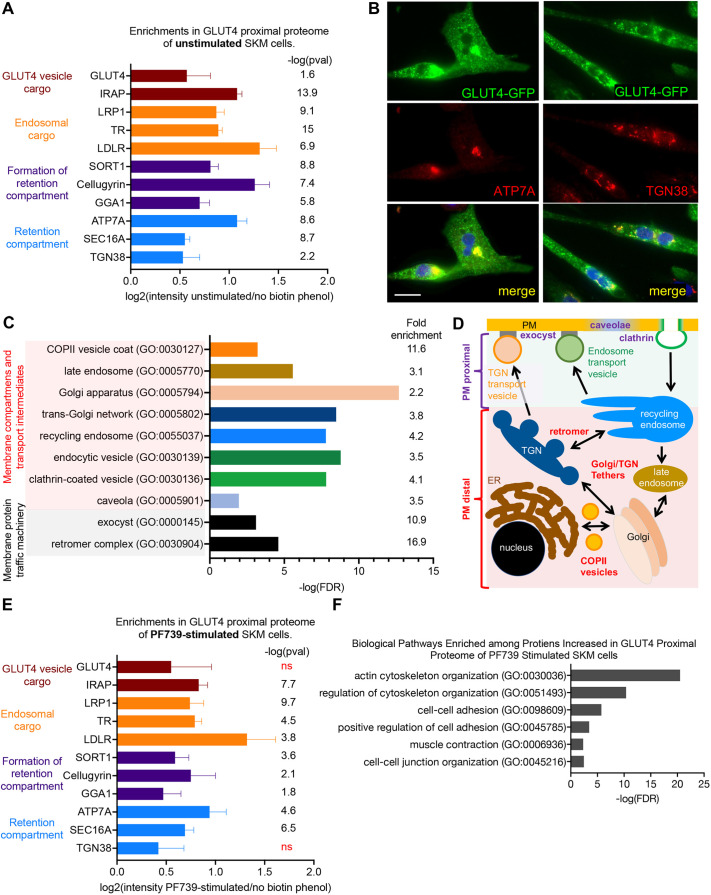
**APEX2 proximal proteome mapping of GLUT4 in unstimulated and PF739-stimulated differentiated SKM cells.** (A) Enrichments of selected proteins in the unstimulated GLUT4-proximal proteome. Log_2_ values of the ratio of mass spectrometry intensities from experimental conditions to mass spectrometry intensities of reactions without biotin–phenol, the APEX2 substrate. The *P*-values [shown as negative log-transformed values; −log(pval)] are significant when tested with Benjamini–Hochberg correction for false discovery at α=0.05. Data are mean+s.e.m. from six experiments. (B) Immunofluorescence colocalizations of ATP7A and TGN38 with HA–GLUT4–GFP in day 3-differentiated SKM cells. Scale bar: 50 µm. Images are representative of two experiments. (C) Some biological groupings, and their associated Gene Ontology (GO) identifiers, significantly overrepresented among proteins of the GLUT4-proximal proteome in unstimulated cells. FDR calculated at α=0.05. Ontology pathway enrichment analyses were performed using Panther online software (Ashburner et al., 2000; Mi et al., 2019a,b). (D) Schematic of compartments and protein complexes represented in the GLUT4-proximal proteome of SKM cells. See text for description. ER, endoplasmic reticulum. (E) Enrichments of selected proteins in the PF739-stimulated GLUT4-proximal proteome. Log_2_ values of the ratio of mass spectrometry intensities from experimental conditions to mass spectrometry intensities of reactions without biotin–phenol, the APEX2 substrate. The *P*-values are significant when tested with Benjamini–Hochberg correction for false discovery at α=0.05. ns, not significant. Data are mean+s.e.m. from six experiments. (F) Some biological groupings, and their associated GO identifiers, significantly overrepresented among proteins increased in the GLUT4-proximal proteome of PF739-stimulated cells compared to that of unstimulated cells. FDR calculated at α=0.05. Ontology pathway enrichment analyses were performed using Panther online software (Ashburner et al., 2000; Mi et al., 2019a,b).

As anticipated, GLUT4 was identified as an APEX2 biotinylated protein enriched over the negative control in all six experiments (Fig. 3A). The transmembrane insulin-regulated aminopeptidase (IRAP, also known as LNPEP) was also identified as a component of the GLUT4-proximal proteome (Fig. 3A). IRAP is the best described cargo of the insulin-regulated transport pathway that traffics GLUT4 to the PM in adipocytes and muscle cells (Descamps et al., 2020). In addition, the endosomal cargo proteins TR, low-density lipoprotein receptor (LDLR) and low-density lipoprotein receptor-related protein 1 (LRP1) were identified as components of the GLUT4-proximal proteome (Fig. 3A). All of these endosomal proteins have been described to localize, to varying degrees, with GLUT4 in adipocytes, despite these proteins not being primary cargos of the insulin-regulated pathway. The proximity of these proteins to GLUT4 detected by APEX2 mapping likely reflects the transit of GLUT4 through common endosomes during constitutive traffic to and from the PM in unstimulated SKM cells. Proteins previously shown to colocalize with GLUT4 in the adipocyte Golgi/TGN retention compartment were significantly enriched in the basal GLUT4-proximal proteome, including those functionally required for GLUT4 traffic in adipocytes – sortilin (Shi and Kandror, 2005, 2007; Shi et al., 2008), cellugyrin (also known as synaptogyrin-2) (Kupriyanova and Kandror, 2000), GGA1 (Watson et al., 2004; Shi et al., 2008) and SEC16A (Bruno et al., 2016; Brumfield et al., 2021) – as well as those with no known function in GLUT4 trafficking – ATP7A (Fazakerley et al., 2015; Brumfield et al., 2021) and trans-Golgi network protein 2 (TGN38) (Fazakerley et al., 2015; Brumfield et al., 2021) (Fig. 3A). Colocalization of GLUT4 with TGN38 and ATP7A were confirmed by immunofluorescence (Fig. 3B). The overlap between GLUT4-proximal proteins in SKM muscle cells with those previously identified to colocalize with GLUT4 in adipocytes and rodent muscle cells supports the hypothesis that GLUT4 traffic pathway is similar between muscle and adipocytes, and to a large degree, conserved between mouse and human cells.

Protein ontology pathway analyses (Ashburner et al., 2000; Mi et al., 2019a,b) established an enrichment of different intracellular compartments and transport pathways among the 700 proteins of the GLUT4-proximal proteome in unstimulated muscle cells (Fig. 3C,D). These data reveal that in unstimulated SKM cells, GLUT4 was widely distributed among many intracellular compartments, providing independent support for our hypothesis that intracellular retention of GLUT4 is dynamically achieved in human muscle cells by constitutive transport of GLUT4 through various intracellular compartments, rather than by static sequestration within a single retention compartment. In addition, there were significant enrichments of cytoskeletal pathways, predominantly those involving the actin cytoskeleton, in the basal proteome (Fig. S3E). Numerous previous studies have documented a role for the actin cytoskeleton in regulation of GLUT4 traffic in rodent muscle cells (reviewed in Sylow et al., 2021).

### GLUT4 translocation downstream of activated AMPK impacts the GLUT4-proximal proteome

We next explored how the GLUT4-proximal proteome is affected by AMPK activation. We focused on AMPK activation, rather than insulin stimulation, given the robust GLUT4 translocation to the PM induced by PF739 as an AMPK activator and the desire to gain mechanistic insights into the AMPK-mediated upregulation of glucose uptake that was previously less well understood. We stimulated SKM cells expressing HA–GLUT4–APEX2 for 90 min with 3 µM PF739, either in the presence of biotin–phenol and H_2_O_2_ (full reaction) or without biotin–phenol (negative control). Of the proteins subsequently isolated by streptavidin precipitation, 399 had increased intensity in the full reaction relative to the negative control [log_2_(intensity full reaction/intensity no biotin–phenol)>0] and a Benjamini–Hochberg false discovery-corrected *P*-value of less than 0.05 (*P*_adj_<0.05) (Table S2). All of these proteins were part of the GLUT4-proximal proteome of unstimulated cells. These data demonstrate that, despite the markedly different kinetics of GLUT4 trafficking between unstimulated and PF739-stimulated cells, GLUT4 traverses many, if not all, of the same intracellular compartments in both states, thereby supporting the hypothesis that redistribution of GLUT4 to the PM is primarily achieved by altering traffic among these compartments rather than mobilizing a pool of GLUT4 that is static in unstimulated cells. Of note, IRAP; the endosomal proteins LRP1, TR and LDLR; and proteins associated with the perinuclear GLUT4-retention compartment (ATP7A, SEC16A, sortilin, GGA1 and cellugyrin) were all in the GLUT4-proximal proteome of cells with activated AMPK (Fig. 3E).

To quantify the effects of activated AMPK on relative abundances of proteins within the GLUT4 proteome, we contrasted the APEX2 mapping results of PF739-stimulated cells to the APEX2 mapping generated in unstimulated cells. Of the 700 proteins of the GLUT4-proximal proteome in unstimulated cells, 526 were less abundant in the PF739-stimulated GLUT4 -proximal proteome [log_2_(intensity in PF739-stimulated cells/intensity in unstimulated cells)<0], and 174 were more highly enriched [log_2_(intensity in PF739-stimulated cells/intensity in unstimulated cells)>0] (Table S3). Proteins involved in the control of actin cytoskeleton dynamics and functions, cell adhesion and cell–cell junction organization were significantly enriched among these 174 proteins (Fig. 3F).

**Fig. 4.**
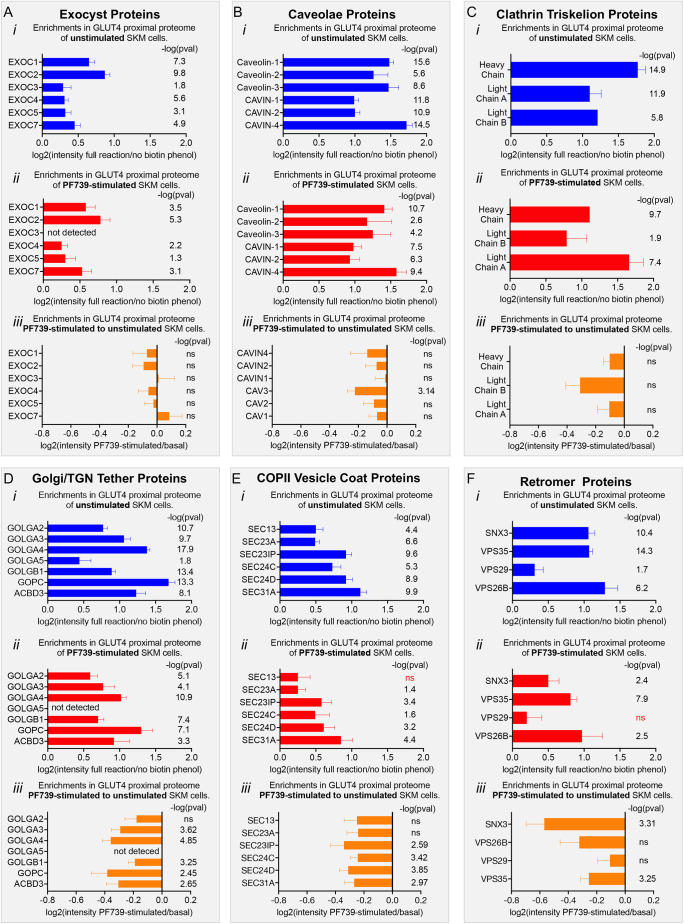
**Impact of PF739 activation of AMPK on components of the GLUT4-proximal proteome.** (A) Exocyst proteins, (B) caveolar proteins, (C) clathrin triskelion proteins, (D) Golgi/TGN tether proteins, (E) COPII vesicle proteins and (F) retromer proteins. Graphs show log_2_ of mass spectrometry ratios for (i) unstimulated to no biotin–phenol negative control, (ii) PF739-stimulated to no biotin–phenol negative control and (iii) PF739-stimulated to unstimulated (basal), calculated for each protein from six experiments. Data are presented as mean+s.e.m. The *P*-values [shown as negative log-transformed values; −log(pval)] are significant when tested with Benjamini–Hochberg correction for false discovery at α=0.05 (ns, not significant at *P*<0.05).

To further explore how the distribution of GLUT4 among the intracellular compartments was affected by PF739-mediated activation of AMPK, we focused on protein complexes and groups known to be involved in membrane protein trafficking that were enriched in the GLUT4-proximal proteome of unstimulated cells (referenced to the no biotin–phenol control): exocyst proteins (Fig. 4Ai), caveolar proteins (Fig. 4Bi), clathrin triskelion proteins (Fig. 4Ci), Golgi/TGN peripheral proteins (Fig. 4Di), COPII vesicle proteins (Fig. 4Ei) and retromer complex proteins (Fig. 4Fi). In each instance, multiple proteins of these complexes or groups were significantly enriched, reinforcing the conclusion that GLUT4 is in proximity to these complexes in unstimulated cells. These complexes and groups were also enriched in the GLUT4-proximal proteome generated in cells stimulated with PF739 (referenced to the no biotin–phenol control) (Fig. 4Aii, Bii, Cii, Dii, Eii and Fii). The proximity of GLUT4 to these proteins in both stimulated and unstimulated cells suggests that they participate in GLUT4 trafficking in both states.

To explore whether the proximities of these proteins to GLUT4 were affected by AMPK activation, we contrasted the APEX2 biotinylation data from cells stimulated with PF739 to data from unstimulated cells. The mass spectrometry intensities of the exocyst (Fig. 4Aiii), caveolar (Fig. 4Biii) and clathrin triskelion proteins (heavy and light chains) (Fig. 4Ciii) were not significantly different in PF739-stimulated SKM cells compared to their intensities in unstimulated SKM cells, indicating that AMPK activation and altered kinetics of GLUT4 trafficking had little effect on the proximity of these protein complexes to GLUT4. The PF739-stimulated to unstimulated ratios of mass spectrometry intensities (log_2_) for Golgi/TGN tether proteins (Fig. 4Diii), COPII vesicle proteins (Fig. 4Eiii) and retromer proteins (Fig. 4Fiii) were all negative, supporting the hypothesis that upon activation of AMPK, GLUT4 moves away from these proteins coincident with GLUT4 redistribution to the PM.

### Proximity proteome of the GLUT4 trafficking mutant F^5^Y-GLUT4 is unaltered by activation of AMPK

A GLUT4 mutant in which the phenylalanine of the amino-terminal cytoplasmic domain FQQI motif (amino acids 5–8) has been mutated to tyrosine displays enhanced intracellular retention and a blunted insulin-stimulated translocation to the PM of adipocytes (Govers et al., 2004; Blot and McGraw, 2008a,b; Brumfield et al., 2021). Despite the increased intracellular retention of F^5^Y-GLUT4, it is in equilibrium with the PM of unstimulated fat cells (Blot and McGraw, 2008a,b; Brumfield et al., 2021). These data support a model in which the tyrosine substitution precludes insulin-stimulated mobilization of F^5^Y-GLUT4 to the PM by biasing F^5^Y-GLUT4 to the basal trafficking itinerary that is unaltered by insulin. We have previously used F^5^Y-GLUT4 as a tool to study GLUT4 trafficking in unstimulated adipocytes (Brumfield et al., 2021).

**Fig. 5.**
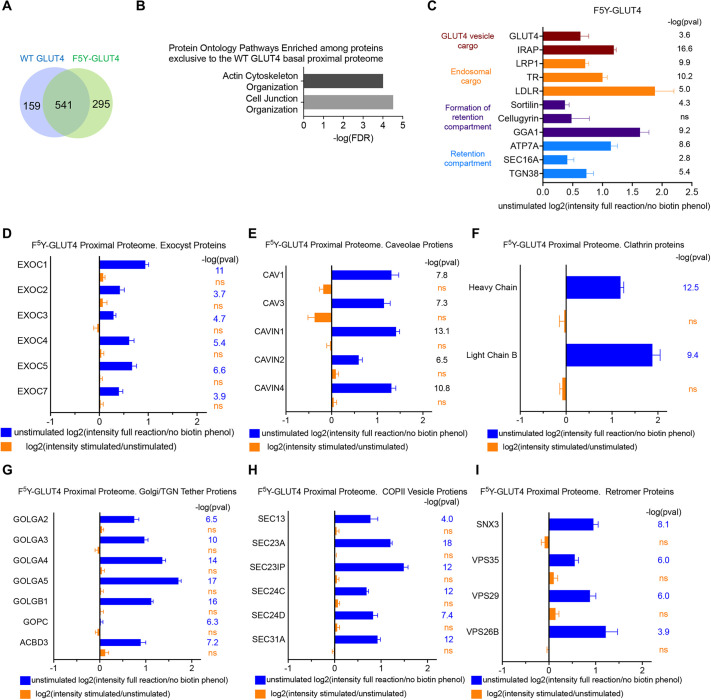
**Impact of F^5^Y mutation in GLUT4, which affects GLUT4 trafficking, on the GLUT4-proximal proteome.** (A) Venn diagram comparing the protein compositions of the unstimulated proximal proteomes of WT GLUT4 and F^5^Y-GLUT4, with numbers of proteins indicated. (B) Some biological groupings, and their associated Gene Ontology (GO) identifiers, significantly overrepresented among proteins in the unstimulated WT GLUT4-proximal proteome that are not represented in the unstimulated F^5^Y-GLUT4-proximal proteome. FDR calculated at α=0.05. Ontology pathway enrichment analyses were performed using Panther online software (Ashburner et al., 2000; Mi et al., 2019a,b). (C) Enrichments of selected proteins in the unstimulated F^5^Y-GLUT4-proximal proteome. Log_2_ values of the ratio of mass spectrometry intensities from experimental conditions to mass spectrometry intensities of reactions without biotin–phenol, the APEX2 substrate. The *P*-values [shown as negative log-transformed values; −log(pval)] are significant when tested with Benjamini–Hochberg correction for false discovery at α=0.05. Data are mean+s.e.m. from six experiments. (D–I) Graphs showing log_2_ mass spectrometry ratios for (D) exocyst proteins, (E) caveolar proteins, (F) clathrin triskelion proteins, (G) Golgi/TGN tether proteins, (H) COPII vesicle proteins and (I) retromer proteins in the F^5^Y-GLUT4-proximal proteome. Log_2_ mass spectrometry ratios for unstimulated to no biotin–phenol negative control (blue) and PF739-stimulated to unstimulated (orange) calculated for each protein from six experiments are shown. Data are mean+s.e.m. The *P*-values shown are significant when tested with Benjamini–Hochberg correction for false discovery at α=0.05. ns, not significant.

HA–F^5^Y-GLUT4–APEX2 (hereafter referred to as F^5^Y-GLUT4) stably expressed in SKM cells was better excluded from the PM than was similarly tagged wild-type GLUT4 (hereafter referred to as WT GLUT4); moreover, AMPK-induced translocation of F^5^Y-GLUT4 to the PM was blocked (Fig. S3D). Thus, the impact of the F^5^Y mutation on GLUT4 behavior in SKM muscle cells is similar to that in adipocytes: enhanced intracellular retention in unstimulated cells and blunted signal-induced translocation. We next generated a F^5^Y-GLUT4-proximal proteome in unstimulated and PF739-treated SKM cells. Analyses of the mass spectrometry intensities from six independent experiments yielded 836 proteins in unstimulated conditions with increased intensity in the biotin–phenol condition relative to the negative control [log_2_(intensity full reaction/intensity no biotin–phenol)>0] and a Benjamini–Hochberg false discovery-corrected *P*-value of less than 0.05 (*P*_adj_<0.05) (Table S4). There was extensive overlap between the unstimulated proximal proteomes of F^5^Y-GLUT4 and WT GLUT4 (Fig. 5A).

Protein pathway ontology of the 295 proteins that were in the F^5^Y-GLUT4-proximal proteome but not the WT GLUT4-proximal proteome did not reveal new compartments or reflect any biology specific to F^5^Y-GLUT4 (Fig. S3F). These proteins were mostly within the same biology groupings as those identified for the WT GLUT4-proximal proteome, reinforcing the hypothesis that F^5^Y-GLUT4 is sequestered within the same compartments as WT GLUT4. Interestingly, there was an enrichment of actin cytoskeleton and cell junction proteins among the proteins in the WT GLUT4- but not F^5^Y-GLUT4-proximal proteome (Fig. 5B). These findings are consistent with the increased WT GLUT4 in the PM and corresponding reduced concentration in the Golgi/TGN compartments compared to that of F^5^Y-GLUT4, which would increase the proximity of WT GLUT4 to actin and junctional proteins at or near the PM.

At the individual protein level, proteins enriched in GLUT4 vesicles and GLUT4-containing compartments in unstimulated SKM cells were similarly enriched in the F^5^Y-GLUT4-proximal proteome (Fig. 5C). In addition, the exocyst, caveolar, clathrin, Golgi/TGN tether protein, COPII vesicle coat and retromer protein complexes and groups, which were enriched in the proximal proteome of WT GLUT4, were similarity enriched in the F^5^Y-GLUT4-proximal proteome of unstimulated SKM cells (Fig. 5D–I). The enrichments of exocyst, caveolar and clathrin proteins in the F^5^Y-GLUT4-proximal proteome were not significantly reduced by AMPK stimulation (Fig. 5D–F; Tables S5, S6), as was the case in the WT GLUT4-proximal proteome. However, unlike the case for WT GLUT4, the enrichments of components of the perinuclear region of cells – Golgi/TGN tethers, COPII vesicle coat and retromer proteins – in the F^5^Y-GLUT4-proximal proteome were not reduced by AMPK stimulation (Fig. 5G–I). These data reinforce our hypothesis that translocation of GLUT4 to the PM primarily involves a redistribution from the Golgi/TGN perinuclear region of cells, since F^5^Y-GLUT4 is neither redistributed to the PM nor translocated away from the Golgi/TGN perinuclear region of the cells.

## DISCUSSION

Despite important advances dissecting the molecular mechanisms regulating GLUT4 traffic to and from the PM of adipocytes and muscle cells, critical features of GLUT4 cell biology and its regulation by metabolic signaling pathways remain incompletely understood. One challenge in studying the cell biology of GLUT4 is that its intracellular retention, translocation and subsequent sequestration following removal of the signal involves a number of cellular compartments, many of which are not specific to GLUT4 trafficking but are common to other transport processes. A detailed understanding of the itinerary of GLUT4 and the compartments traversed, both those common to other pathways and those more restricted to GLUT4, is required to understand molecular mechanisms that regulate the amount of GLUT4 in the PM, and consequently for a more complete description of the role of GLUT4 in whole-body metabolic regulation. APEX2 mapping provides a comprehensive view of the location of a protein. The GLUT4-proximal proteomes we have developed provide novel, comprehensive, high-resolution (∼20 nm) views of the GLUT4 trafficking itinerary in human skeletal muscle cells and of how that itinerary is altered upon AMPK-induced GLUT4 translocation to the PM. This information on its own advances the field of GLUT4 cell biology by providing unbiased data in support of the dynamic retention model of GLUT4 retention and redistribution. In addition, the proximal proteome maps are a foundation for future studies of the molecular mechanisms controlling GLUT4 traffic.

IRAP is known to follow the same trafficking pathway as GLUT4 in adipocytes and muscle cells, and its enrichment in the GLUT4-proximal proteome provides validation for the data set. In addition, proteins previously shown to be required for generation of GLUT4 retention in unstimulated rodent adipocytes (sortilin, GGA1 and cellugyrin), were enriched in the GLUT4-proximal proteome of unstimulated human muscle cells. Other proteins known to localize with or near GLUT4 in adipocytes – ATP7A, SEC16A and TGN38 – were also enriched in the proximal proteome of unstimulated human muscle cells. Thus, our novel findings reveal extensive overlap in the trafficking itinerary of GLUT4 (defined by the proximal proteome) from rodents to humans, and between adipocytes and muscle cells.

From a mechanistic perspective, the diversity of endomembrane compartments represented in the GLUT4-proximal proteome of unstimulated human muscle cells supports a dynamic intracellular retention model in which GLUT4 continually recycles between the PM and intracellular compartments. The steady state accumulation in the PM reflects the overall rates of exocytosis and endocytosis, rather than static sequestration by tethering of GLUT4 within cells in the absence of signaling. The impact of AMPK activation on the GLUT4-proximal proteome further supports the redistribution among compartments accessible in unstimulated cells. Our dynamic retention and redistribution model is also supported by our kinetic studies, in which we find constitutive endocytosis and exocytosis of GLUT4 in unstimulated and stimulated SKM cells. We have proposed a similar model for GLUT4 trafficking in adipocytes (Karylowski et al., 2004; Martin et al., 2006; Bruno et al., 2016; Brumfield et al., 2021). Our data supporting dynamic retention of GLUT4 in unstimulated human muscle cells agrees with results of studies of rodent muscle cells (Li et al., 2001; Randhawa et al., 2004; Klip et al., 2019; Sylow et al., 2021). There are considerable precedents for steady state concentrations of membrane proteins within organelles and membrane compartments by active retrieval mechanisms, as we propose for GLUT4 (Banfield, 2011). However, an alternative model in which intracellular GLUT4 is not in equilibrium with the PM of unstimulated adipocytes and muscle cells and only traffics to the PM upon stimulation has been proposed (Coster et al., 2004; Govers et al., 2004; Bogan and Kandror, 2010; Meriin et al., 2022). Our kinetic data and APEX2 mapping data do not support a static retention model.

We establish that AMPK activation induces GLUT4 translocation in human muscle cells by inhibition of endocytosis and acceleration of exocytosis. These data identify potential differences between humans and rodents because it has previously been shown that AMPK activation (using either AICAR or the protonophore 2,4-dinitrophenol) induces GLUT4 translocation in L6 rat muscle cells by inhibition of endocytosis (Antonescu et al., 2008; Fazakerley et al., 2010). In addition, we have also shown that the TBC1D4–Rab10 signaling module, which is key for insulin-induced translocation of GLUT4 to the PM of adipocytes, is required for AMPK-induced GLUT4 translocation in human muscle cells. Thus, the model we developed here can be used to further dissect the molecular mechanism linking physiologic signaling to the control of glucose uptake in human muscle cells.

Previous studies in adipocytes and rodent muscle cells have identified roles for the exocyst (Inoue et al., 2003; Fujimoto et al., 2019; Wang et al., 2019), caveolae (Kandror et al., 1995; Fecchi et al., 2006; Gonzalez-Munoz et al., 2009), clathrin triskelion proteins (Antonescu et al., 2008; Bogan and Kandror, 2010) and retromer (Yang et al., 2016; Pan et al., 2017) in the control of GLUT4 trafficking in fat cells. Enrichment of these proteins in the GLUT4-proximal proteome of unstimulated cells highlights potential roles of the membrane transport steps controlled by these proteins in the steady state intracellular retention of GLUT4 in human muscle cells. The enrichment of COPII vesicle proteins and Golgi proteins suggests that in unstimulated muscle cells, GLUT4 cycles through or near both PM-proximal compartments and more PM-distal compartments (ER–Golgi/TGN region). These data, particularly the GLUT4 proximity to COPII vesicle coat proteins, agree with the hypothesized role for the ER–Golgi intermediate compartment (ERGIC) in GLUT4 retention in muscle cells (Camus et al., 2020). However, it is important to note that the proximity of GLUT4 to COPII vesicles does not mean that GLUT4 is transported by COPII vesicles. Clathrin, in addition to having a role in GLUT4 internalization, has been reported to have a specific role in intracellular traffic of GLUT4 in human muscle, controlled by the human-specific clathrin heavy chain isoform CHC22 (also known as CLTCL1) (Gould et al., 2020); however, our analyses of the mass spectrometry data do not distinguish between CHC22 and the more conventional clathrin heavy chain CHC17 (also known as CLTC).

Analyses of how AMPK activation alters the enrichment of these complexes in the GLUT4-proximal proteome revealed no significant change for the exocyst, caveolar and clathrin triskelion proteins, all of which are associated with more peripheral trafficking steps. Strikingly, enrichment of the more PM-distal aspects of cycling to and from the cell surface was reduced upon AMPK stimulation, including that of retromer, Golgi tethers and COPII vesicle proteins. These data support a model in which AMPK-dependent acceleration of GLUT4 exocytosis is accompanied by a depletion of GLUT4 from the more PM-distal compartments (that is, Golgi/TGN-proximal compartments) without a significant depletion from the PM-proximal ones (Fig. 4D). This link between reduced traffic of GLUT4 through Golgi-proximal pathways and increased PM expression is also supported by the proximal proteome of F^5^Y-GLUT4. In this case, the blunted AMPK-induced PM translocation of F^5^Y-GLUT4 was associated with no redistribution of GLUT4 away from the Golgi-proximal compartments. Reduced GLUT4 proximity to retromer, Golgi/TGN tether and COPII vesicle proteins in cells with activated AMPK is consistent with a more prominent role for these complexes in basal state retention. Nonetheless, they were also components of the GLUT4-proximal proteome of stimulated cells, establishing a similar GLUT4 itinerary in unstimulated and stimulated cells. Additional studies are required to functionally test the models of GLUT4 trafficking in human muscle cells that emerged from the APEX mapping data.

The most pronounced difference between the unstimulated and AMPK-stimulated GLUT4-proximal proteomes was an increase in proteins involved in regulation of the actin cytoskeleton. These differences likely reflect a role for actin in the enhanced movement of GLUT4 to the PM of muscle cells, consistent with previous functional studies in rodent muscle cell lines (Tong et al., 2001; Talior-Volodarsky et al., 2008; Moller et al., 2019). The fewer proteins involved in actin biology in the F^5^Y-GLUT4-proximal proteome of unstimulated cells also highlights the role of the actin cytoskeleton in the constitutive transport of GLUT4 to the PM. The GLUT4-proximal proteome of PF739-stimulated cells did not support localization of GLUT4 to specific PM domains (or proximity to specific proteins) within the PM that might be linked to its function in increased glucose uptake, although such regulation might not be captured in the cultured cell model used.

The TBC1D4–Rab10 signaling module is required for insulin-stimulated GLUT4 translocation in rodent adipocytes, with TBC1D4 promoting intracellular retention by inhibiting Rab10, and Rab10 promoting insulin-stimulated translocation (Klip et al., 2019). GLUT4 exocytosis is the target of TBC1D4–Rab10. Here, we show that the TBC1D4–Rab10 signaling module is also required for regulation of GLUT4 traffic in human muscle cells: TBC1D4 for GLUT4 intracellular retention in the basal state, and Rab10 for GLUT4 translocation to the PM in response to AMPK. A major question in the field is to understand the impact of muscle contraction/exercise and AMPK activation on insulin-stimulated GLUT4 translocation (Jensen and O'Rahilly, 2017). Although the data presented here do not specifically address that question, the novel detailed information on GLUT4 itineraries in unstimulated and AMPK-stimulated cultured human muscle cells provide a foundation for future functional and mechanistic studies interrogating the intersection of signal transduction downstream of AMPK and the insulin receptor.

## MATERIALS AND METHODS

### Chemicals and drugs

Chemicals and drugs used were PF739 (Pfizer), Dynasore (S8047, Selleck Chemicals), insulin (I5500, Sigma-Aldrich) and AICAR (M1404, Sigma-Aldrich).

### Cell culture and differentiation of human skeletal muscle cells

SKM cells, an immortalized human muscle cell line derived from GIBCO human skeletal myoblast cells (Thermo Fisher Scientific, A11440) by stable lentiviral transduction with hTERT and CDK4 (Cokorinos et al., 2017), were grown in muscle cell growth medium (C-23060*,* PromoCell) supplemented with 2% horse serum (16050122, Thermo Fisher Scientific), 1% chick embryo extract (C3999, US Biologicals) and penicillin/streptomycin (15070063, Thermo Fisher Scientific) at 37°C and 5% CO_2_. For differentiation, SKM cells were grown to confluency, the medium switched to differentiation medium containing Dulbecco's Modified Eagle's Medium low glucose, pyruvate (DMEM; 11885092, Thermo Fisher Scientific), supplemented with 2% horse serum and penicillin/streptomycin. Unless noted otherwise, cells were studied at day 3 post-differentiation.

### Generation of stable expression and CRISPR-Cas9 knockout cell lines

Engineered SKM cell lines were generated via lentiviral transduction. HEK293T packaging cells (ATCC CRL-3216) were transfected with the required lentiviral cDNA constructs using the Lenti-X packaging system following the manufacturer’s protocol (631276, Takara). SKM cells stably expressing HA–GLUT4–GFP were generated by transducing with HA–GLUT4–GFP in pLenti6 lentiviral vector (K4955-10 Life Technologies; Brumfield et al., 2021). Following growth, cells were sorted for GFP to isolate a pooled population of SKM cells stably expressing HA–GLUT4–GFP (referred to as SKM-GLUT4 cells). SKM-GLUT4 cells stably expressing CRISPR-Cas9 (SKM-GLUT4-CRISPR-Cas9) were generated by transducing SKM-GLUT4 with CRISPR-Cas9 lentivirus (A32069, Thermo Fisher Scientific). Transduced cells were selected for stable CRISPR-Cas9 expression using hygromycin selection. Stable expression of CRISPR-Cas9 did not affect cell differentiation nor response to PF739 (data not shown). Rab10-knockout and TBC1D4-knockout cells were generated by transducing SKM-GLUT4-CRISPR-Cas9 cells with lentivirus containing gRNAs targeting Rab10 or TBC1D4, respectively, followed by Zeomycin selection to enrich for cells expressing gRNAs. SKM-GLUT4-CRISPR-Cas9 cells were used as controls in studies of Rab10- and TBC1D4-knockout lines. gRNA sequences were as follows: Rab10, 5′-CACCGCATTGCGCCTCTGTAGTAGG-3′; TBC1D4, 5′-CACCGGAAACAGGCCTTCAGTACGG-3′.

### siRNA knockdown in SKM cells

SKM myotubes at day 3 of differentiation (one well of a six-well cluster) were trypsinized, pelleted, resuspended in 500 µl serum-free DMEM medium with penicillin/streptomycin, mixed with 2 nM of siRNA and electroporated at 180 V and 950 µF. Electroporated cells were plated in SKM differentiation medium on coverslips, incubated for 3–4 h in a 37°C and 5% CO_2_ incubator, followed by addition of 2 ml of SKM differentiation medium. After 48–72 h, cells were used for translocation assays, and for analyzing mRNA and protein expression. The siRNAs were purchased from Integrated DNA Technologies. The siRNA sequences were as follows: Rab10 si1, 5′-UAUGACAUCACCAAUGGUAAAAGTT-3′; Rab8A si1, 5′-GGCAAGAGAAUUAAACUGCAGAUAT-3′.

### RNA isolation and quantitative real-time polymerase chain reaction

mRNA was isolated using an Rneasy kit (74106, QIAGEN), and cDNA was prepared using an RNA to cDNA EcoDry Premix (639545, Takara Bio). Quantitative real-time PCR was performed using appropriate primer pairs from the PrimerBank database (https://pga.mgh.harvard.edu/primerbank/). Primers were as follows. Human Rab10: forward, 5′-CTGCTCCTGATCGGGGATTC; reverse, 5′-TGATGGTGTGAAATCGCTCCT-3′. Human TBC1D4: forward, 5′-AGATGGCCTGCCACGTTTT-3′; reverse, 5′-GGCCGCTTTAGATAATTGCCTTA-3′. Human Rab8A: forward, 5′-CGAAGGCCAACATCAATGTGG-3′; reverse, 5′-TCCGGTGTGATTTTGACTCCC-3′. Human GAPDH: forward, 5′-CTTCAACAGCGACACCCACTCCTC-3′; reverse, 5′-GTCCACCACCCTGTTGCTGTAG-3′.

### Western blot analysis

Proteins were extracted from 3-day differentiated SKM cells using cell lysis buffer (9803S, Cell Signaling Technology), supplemented with a complete protease and phosphatase inhibitor cocktail (78442, Thermo Fisher Scientific) and boiled at 95°C for 5 min. Proteins were displayed on SDS-PAGE gels (8, 10 or 12% acrylamide, depending on the molecular mass of the protein to be visualized), transferred onto nitrocellulose membranes and blocked at room temperature for 1 h in 5% milk or 5% BSA (for labelled-antibodies) in Tris-buffered saline with 0.5% Tween-20 (TBS-T). Membranes were then incubated with primary antibodies (diluted in 5% milk or 2.5% BSA with TBS-T) overnight at 4°C. Primary antibodies used for western blotting were against β-tubulin (1:2000; ab6046, Abcam), Rab10 (1:500; 4262S, Cell Signaling Technology), Rab8A (1:1000; 610844, BD Biosciences), myosin heavy chain (1:1000; M4276, Millipore Sigma), myogenin (1:1000; ab1835, Abcam), total acetyl-CoA carboxylase (1:1000; 3676, Cell Signaling Technology), labelled-acetyl-CoA carboxylase (Ser79 ACC) (1:500; 3661, Cell Signaling Technology), labelled T308-AKT (1:500; 2965S, Cell Signaling Technology), total AKT (1:1000; 9272S, Cell Signaling Technology), labelled-T642 TBC1D4 (1:1000; 8881S, Cell Signaling Technology) and total TBC1D4 (1:1000; 07741, Millipore Sigma). Blots were incubated with goat anti-rabbit IgG­ HRP-conjugated or anti-mouse IgG HRP-conjugated secondary antibody (diluted 1:5000 in 5% milk or 2.5% BSA with TBS-T). Blots were visualized using My ECL Imager (Thermo Fisher Scientific) and analyzed using ImageStudioLite (Li-Cor Biosciences). Tubulin expression was used as a loading control where indicated.

### Microscopy

Images were acquired on an inverted epifluorescence microscope using a 20× air objective or a 40× oil objective (Leica Biosystems). Images were analyzed on MetaMorph (Universal Imaging) image processing software by appropriate thresholding and quantification, as previously described (Lampson et al., 2001). Signals for GFP and Cy3 were corrected for background, and the surface (Cy3) to total (GFP) GLUT4 (S:T) was calculated per cell area, as previously detailed (Lampson et al., 2001). Airyscan images to acquire *Z* stacks of the cells were collected using a laser scanning microscope (LSM880, ZEISS) with Airyscan using a 63×oil objective.

### GLUT4 translocation assay in SKM cells

HA–GLUT4–GFP translocation assay was performed as described previously (Lampson et al., 2001) with the following modifications. SKM cells at day 3 of differentiation stably expressing HA–GLUT4–GFP were incubated in serum-free medium for 2 h. Cells were stimulated with insulin or PF739 as specified in each experiment. Cells were then fixed in 3.7% formaldehyde for 6 min, and PM HA–GLUT4–GFP was detected using an anti-HA antibody (1:1000; 901503, BioLegend). HA staining was visualized by Cy3–goat anti-mouse IgG secondary antibody (1:300; 115-165-062, Jackson Immunoresearch). Cell nuclei were labelled with DAPI. Total HA–GLUT4–GFP was visualized by direct fluorescence of GFP. PM to total ratiometric values (dimensionless) were calculated by contrasting the PM HA signal intensity to the GFP signal per cell, after background correction for each channel (Lampson et al., 2001). The mean PM to total resulting values of 40–70 cells per condition were averaged, and those averages across multiple independent experiments were compared. For graphical presentation of data across experiments, the values from individual experiments were normalized to a condition common to each experiment (for example, PM to total ratio of control unstimulated cells). Statistics were performed on non-normalized data.

### Immunofluorescence localization

Fixed and saponin permeabilized SKM-GLUT4 cells where stained with either 1:500 anti-ATP7A (Lifespan Biosciences, LS-C209614) or 1:500 anti-TGN38 (Abcam ab16059) antibodies followed by 1:300 Cy3 goat anti-mouse IgG (115-165-062 Jackson Research).

### Exocytosis assay

Exocytosis assays were carried out as previously described (Blot and McGraw, 2008a,b). Briefly, live cells were incubated in serum-free medium at 37°C for 2 h, following which they were incubated in 2 ml of medium containing insulin or PF739 for 60 min to achieve new steady state of GLUT4 distribution. Cells were then incubated with a saturating concentration of anti-HA antibody (HA.11; 901503, BioLegend) (determined empirically for each batch of HA.11 antibody) in serum-free DMEM supplemented with 1 mg/ml ovalbumin for corresponding time points at 37°C and 5% CO_2_ in the absence (control) or presence of the stimulant. Total cell-associated anti-HA antibody at specified times of incubation were determined in fixed, saponin-permeabilized cells by secondary staining with Cy3–goat anti-mouse IgG antibody. Values of Cy3:GFP were calculated for multiple cells at individual time points and the mean values were plotted in Graphpad Prism and fitted to a one-phase exponential equation. The slope of this curve provided the value of the rate of exocytosis (min^−1^). A separate set of dishes was used to quantify GLUT4 translocation PM-to-total ratio in the absence or presence of the particular stimulant, as a control to verify the effect of the stimulant. Cells were imaged and analyzed as described above.

### Endocytosis assay

GLUT4 endocytosis assay was carried out as previously described (Blot and McGraw, 2008a,b). Briefly, live cells were pre-incubated in serum-free medium at 37°C for 2 h, followed by incubation in 2 ml of medium containing insulin or PF739 for 60 min to achieve new steady-state of GLUT4 distribution. Cells were then incubated with a saturating concentration of anti-HA antibody (HA.11) (determined empirically for each batch of HA.11 antibody) in serum-free DMEM supplemented with 1 mg/ml ovalbumin for corresponding time points at 37°C and 5% CO_2_ in the absence (control) or presence of the particular stimulant. Cells were washed and fixed, and PM-bound HA.11 was stained with a saturating concentration of Cy5–goat anti-mouse IgG antibody (A10523, Life Technologies). Cells were then refixed, permeabilized with saponin and stained with Cy3–goat anti-mouse IgG antibody to reveal internal HA.11 (the Cy3 antibody binds to the same epitopes as the Cy5 antibody, and therefore does not bind to the surface-exposed epitopes already bound by the Cy5 antibody previously). The internal to PM value per cell was calculated for individual cells and averaged for each time point. A plot of that ratio versus time yields a straight-line whose slope is the internalization rate constant (Blot and McGraw, 2008a,b).

TR internalization was assayed by incubating cells in serum-free DMEM containing 3 µg/ml of Cy3-labeled transferrin (made as decribed by Brumfield et al., 2021) for specified times, in the absence (control) or presence of Dynasore (100 µM) or PF739 (3 µM). Following fixation, cells were incubated with a saturating concentration of monoclonal anti-TR antibody (B3/25; ab84036, Abcam) in serum-free DMEM supplemented with 1 mg/ml ovalbumin, for corresponding time points at 37°C and 5% CO_2_. Cells were then washed and fixed, and surface-exposed TR was stained with a saturating concentration of Cy5–goat anti-mouse IgG antibody, without permeabilizing the cells, to label the PM TR.

For both GLUT4 and TR internalization experiments, the internal to PM value per cell (Cy3:Cy5) was calculated for individual cells and averaged for each time point. A plot of that ratio versus time yields a straight-line whose slope is the internalization rate constant (Blot and McGraw, 2008a,b). A separate set of dishes was used to quantify GLUT4 translocation surface-to-total ratio in the absence or presence of the particular stimulant, as a control to verify the effect of the stimulant.

### HA–GLUT4–APEX2

HA–GLUT4–APEX2 was created by replacing the GFP of HA–GLUT4–GFP in pLenti plasmid with APEX2 sequences (Hung et al., 2016), and the resulting construct was confirmed by sequencing. HA–F^5^Y-GLUT4–APEX2 was created using a Quikchange kit (Agilent, Inc) to change Phe5 to Tyr in the GLUT4 sequence of HA–GLUT4–APEX2 in pLenti plasmid.

### HA–GLUT4–APEX2 translocation

Unlike measurement of PM HA–GLUT4–GFP, which is a single-cell ratiometric determination using GFP fluorescence power to normalize the anti-HA signal per cell, measurement of PM HA–GLUT4–APEX2 was a cell population measurement in which the average PM anti-HA staining of cells (fixed, intact cells) from one dish was divided by the total HA–GLUT4–APEX2 expression measured in a separate dish by anti-HA immunofluorescence staining of fixed, permeabilized cells. This was necessary because the APEX2 construct does not have a second tag that can be used to normalize to total expression per cell. Because of needing to use population rather than single-cell normalized PM measures, the net translocation (that is, fold increase of PF739-stimulated over unstimulated) was smaller than that determined using ratiometric (that is, values corrected for expression per cell) measurements, thereby accounting for the apparently smaller translocation of HA–GLUT4–APEX2 than that of HA–GLUT4–GFP.

### APEX2 biotinylation

APEX2 proximity biotinylation assays were performed as previously described (Hung et al., 2016). Briefly, SKM cells at day 3 of differentiation stably expressing HA–GLUT4–APEX2 were incubated in serum-free medium at 37°C for 2 h followed by a 60 min incubation with or without biotin–phenol (500 µM) in serum-free medium. For PF739-stimulated conditions, PF739 was added to a final concentration of 3 µM for the final 30 min of the 2 h incubation in serum-free medium and 3 µM PF739 was maintained in the medium during incubation with biotin–phenol. Following the incubation with biotin–phenol, the cells were rapidly washed, placed on ice and incubated for 1 min with pre-chilled (4°C) 0.34% hydrogen peroxide (H_2_O_2_) in phosphate-buffered saline (PBS). Cells were washed three times with ice-cold quenching solution (5 mM Trolox, 10 mM sodium ascorbate, 10 mM sodium azide in 1x PBS) and lysed in 1× RIPA lysis buffer [5 mM Trolox, 10 mM sodium ascorbate, 10 mM sodium azide, protease and phosphatase inhibitors (1:100, Halt protease and phosphatase inhibitor; 78442, Thermo Fisher Scientific) in H_2_O] before the protein concentration was measured. Cell extracts were tumbled over night at 4°C with 200 µl of Streptavidin agarose beads (Pierce) per 1 mg of cell extract in 500 µl final volume. Beads were washed twice with 1× RIPA lysis buffer, once with 1 M KCl, once with 0.1 M Na_2_CO_3_, once with 2 M urea in 10 mM Tris-HCl (pH 8.0), twice with 1× RIPA lysis buffer, twice with 50 mM Tris-HCl (pH 7.5) and twice with 2 M urea in 50 mM Tris-HCl (pH 7.5).

### Proteomics analyses

#### Digestion and tandem mass tag labelling

Immunoprecipitated samples (in 80 μl of 2 M urea in 50 mM Tris-HCl pH 7.5) were treated with 0.1 M DL-dithiothreitol to a final concentration of 6.25 mM for 30 min at 25°C with shaking on a Thermomixer (Thermo Fisher Scientific). Free cysteine residues were alkylated with 550 mM 2-iodoacetamide at a final concentration of 34 mM for 30 min at 25°C while protected from light. LysC (1 μg; Fujifilm Wako) was added, followed by incubation for 1 h at 25°C with shaking on a Thermomixer. After incubation, the urea concentration was reduced to <1 M with the addition of 150 μl of NH_4_HCO­_3_. Finally, trypsin (2 μg; Pierce) was added, followed by incubation for 16 h at 37°C with shaking on a Thermomixer.

After incubation, the digest was acidified with the addition of 5 μl of formic acid, and samples were brought to 1 ml with 0.1% formic acid. Peptides were desalted on C18 Sep-Pak cartridges (Waters, Milford, MA, USA). Briefly, cartridges were conditioned by sequential addition of (1) methanol, (2) 80% acetonitrile (ACN) with 0.1% formic acid, (3) 0.1% formic acid and (4) 0.1% formic acid. All conditioning volumes were 800 μl, and vacuum was used to elute the solutions. Following conditioning, the acidified peptide digest was loaded onto the cartridge with vacuum. The stationary phase was washed once with 800 μl of 0.1% formic acid. Finally, samples were eluted from the cartridge using 300 μl of 80% ACN with 0.1% formic acid. Eluted peptides were dried under vacuum, followed by reconstitution in water. Peptide yield was quantified by NanoDrop (Thermo Fisher Scientific). An aliquot of 10 μg of peptide per sample was transferred to a fresh Eppendorf tube, followed by chemical tandem mass tag (TMT) labelling with TMTPro 16-plex reagent (Thermo Fisher Scientific). The samples were divided in two TMTPro 16-plexes, with each plex containing three biological replicates per condition. All steps related to TMT labelling were carried out according to the manufacturer's instructions, TMT labelling, quenching and combining of TMT-labelled samples. Once combined, a 20 μg aliquot of combined/labelled peptide material was transferred to a fresh Eppendorf tube and dried under vacuum.

### Basic reversed-phase fractionation and sample loading

The peptide aliquot was reconstituted in 300 μl of 0.1% trifluoroacetic acid, followed by fractionation on a high pH/reversed-phase spin column (Thermo Fisher Scientific) according to the manufacturer's instructions. The resulting eight fractions were dried under vacuum. Each fraction was then reconstituted in 20 μl of 5% ACN with 0.1% formic acid, sonicated and transferred to an autosampler vial. Vials were stored in the autosampler of an Easy-1200 nanoLC (Thermo Fisher Scientific) liquid chromatography (LC) system maintained at 10°C.

### Mass spectrometry analyses

Mass spectrometry analyses used LC–tandem mass spectrometry (MS/MS). Peptides were separated on a 50 cm column composed of C18 stationary phase (Thermo Fisher Scientific, ES903) using a gradient from 5% to 30% buffer B over 2 h (buffer A: 0.1% formic acid in HPLC-grade water; buffer B: 80% ACN, 0.1% formic acid) with a flow rate of 250 nl/min using an EASY-1200 nanoLC system (Thermo Fisher Scientific). MS data were acquired on a QE-HF mass spectrometer (Thermo Fisher Scientific) using a data-dependent acquisition top 10 method, AGC target 5e5, maximum injection time of 50 msec, scan range of 350–1800 *m*/*z* and a resolution of 60 K. MS/MS was performed at a resolution of 60 K, AGC target 1e5, maximum injection time of 105 msec, isolation window 0.7 m/z. Dynamic exclusion was set to 20 s.

All LC-MS/MS runs were analyzed using the Sequest algorithm (SEQUEST HT, Thermo Fisher Scientific) within Proteome Discoverer 2.4 (Thermo Fisher Scientific) against the UniProt human database. A 10 ppm MS1 error tolerance was used. Trypsin was set as the enzyme, allowing for two missed cleavages. TMT tags on lysine residues and peptide N termini (229.162932 Da), and carbamidomethylation of cysteine residues (57.02146 Da) were set as static modifications, while oxidation of methionine residues (+15.99492 Da) and acetylation of peptide N termini (42.011 Da) were set as variable modifications. Percolator was used as the false discovery rate (FDR) calculator, and all peptides were filtered at the ‘strict’ target FDR level of 0.01. Protein assembly was guided by principles of parsimony to produce the smallest set of proteins necessary to account for all observed peptides. Quantitation was performed in Proteome Discoverer with a reporter ion integration tolerance of 20 ppm for the most confident centroid within the ‘Reporter Ions Quantifier’ node, and the MS order was set to MS2. Reporter ion values were corrected for isotopic impurities using the manufacturer provided factors.

The proteomics workflow started from the reporter ion intensities per feature reported in the Proteome Discoverer PSMs.txt file. When a peptide and charge combination was measured multiple times in a sample, only the maximum intensity was kept. The log_2_ peptide intensities were median-normalized assuming equal input loading of all channels. Peptide intensities were summarized to protein intensities using Tukey's median polish algorithm (Huang et al., 2020). Protein normalization using bridge channel was applied afterwards.

Ontology pathway enrichment analyses of the mass spectrometry data were performed using Panther online software (Ashburner et al., 2000; Mi et al., 2019a,b).

### Statistical analysis

#### Mass spectrometry

Outcomes were preprocessed log-transformed proteins. A mixed-effects model with appropriate fixed, random, correlation and variance structure was used for all outcomes. If a random effect was not necessary, a generalized least-squares linear model with appropriate fixed, correlation and variance structure was used. All residuals were evaluated for meeting the normality assumption with the Shapiro–Wilks Test and Q–Q plots and the homogeneity/sphericity assumption by residual plots. The log_2_ ratios of mass spectrometry intensities were tested for multiple testing false discovery using the Benjamini–Hochberg approach. All analyses were performed using R version 3.5.1.

#### GLUT4 trafficking assays

Data analyses were performed using R version 3.5.1 and graphed using Graphpad Prism (v9.5.1). Results are expressed as mean±s.e.m. from at least three independent experiments, in which greater than 40 cells per condition were analyzed. Statistical analyses are specified in the legends. All statistically analyses were done using raw, non-normalized data, although in some instances, for presentation purposes, normalized data are shown. Values of *P<*0.05 were considered to be statistically significant.

## Supplementary Material

10.1242/joces.261454_sup1 Supplementary information

Table S1. WT GLUT4-APEX2 proximal proteome in unstimulated SKM cells.

Table S2. WT GLUT4-APEX2 proximal proteome in PF739 stimulated SKM cells.

Table S3. WT GLUT4-APEX2 proximal proteome in PF739 stimulated SKM cells contrasted to unstimulated cells.

Table S4. F5Y-GLUT4-APEX2 proximal proteome in unstimulated SKM cells.

Table S5. F5Y-GLUT4-APEX2 proximal proteome in PF739 stimulated SKM cells.

Table S6. Effect of AMPK activation of F5Y-GLUT4 unstimulated proximal proteome.
