# Supplementary material for: Regulated dynamic subcellular GLUT4 localization revealed by proximal proteome mapping in human muscle cells
Source: J Cell Sci. 2023 Dec 21;136(23):jcs261454. doi: 10.1242/jcs.261454 (PMC10753500; doi:10.1242/jcs.261454)
Supplement: Supplementary information [file joces-136-261454-s1.pdf]

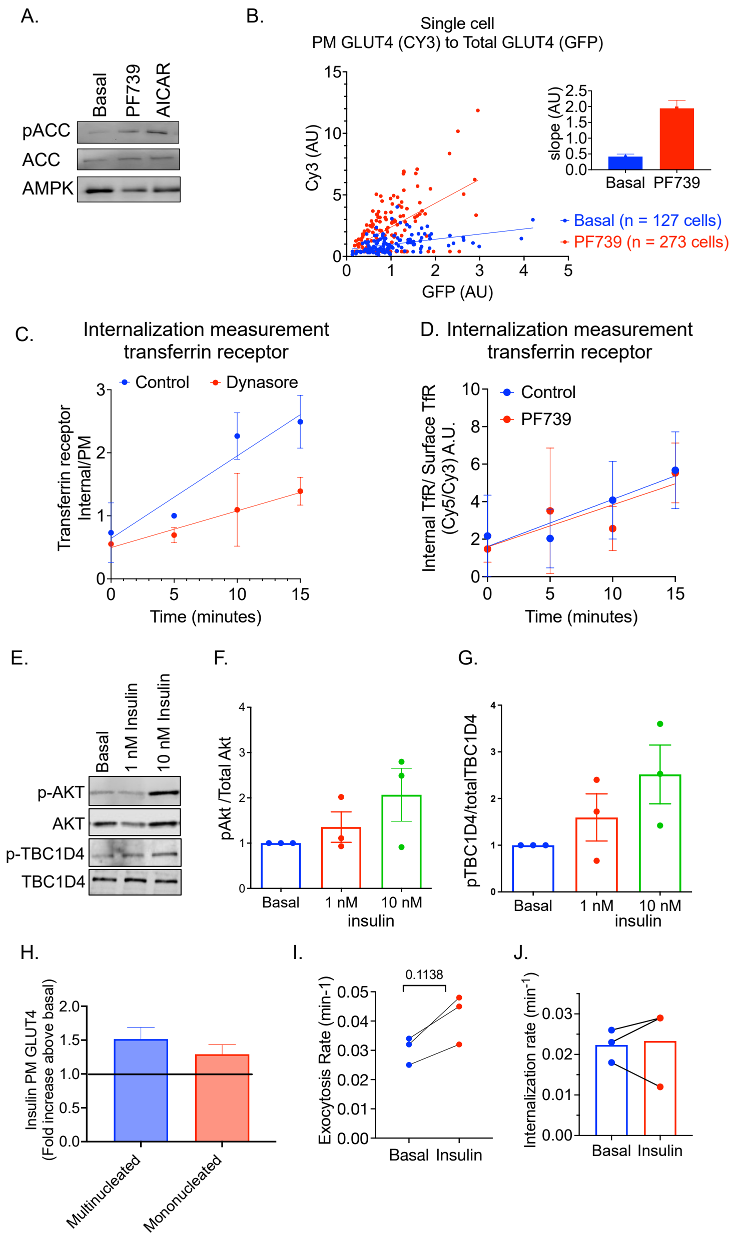

Fig. S1. in support of Figure 1. A. Day-3 differentiated SKM-GLUT4 cells treated for 60 min with vehicle, 3 µM PF739 or 1 mM AICAR blotted for phospho ACC (Ser79), total ACC, and AMPK. **B.** Plot of Cy3 versus GFP per cell. Inset are slopes of the straight line fits of the data, which are proportional to PM-to-total HA-GLUT4-GFP for the population of cells. PF739 stimulation of 60 min at 3 µM. **C.** Effect of 100 µM Dynasore on transferrin receptor (TR) internalization. Internalization rate constant is the slope of the line. Symbols are the mean ± SD of data from 3 independent experiments. Data are normalized to the 5-min data point of the control experiment of the individual experiments. **D.** Effect of 3 µM PF739 on TR internalization. Internalization rate constant is the slope of the line. Data are from a representative experiment. Each symbol is the mean value of at least 40 cells ± SD. **E.** Western blot analysis of pAkt (The308) and pAS160 (T642) in day 3-differentiated SKM-GLUT4 cells under basal and insulin stimulated (1 nM and 10 nM for 30 minutes) conditions. **F., G.** Quantification of phosphoblots from 3 independent experiments like that shown in panel D. ***H.*** Quantification of PM HA-GLUT4-GFP in day-3 differentiated SKM-GLUT4 cells (mononucleated and multinucleated). Serum starved cells were treated without (basal) or with 10 nM insulin for 30 minutes. The mean fold increase in PM HA-GLUT4-GFP above unstimulated cells determined from 40-70 cells per condition were calculated in 16-18 independent experiments. Data are presented as mean ± SEM of the independent experiments. log Transformation Student's 2 sample t-test p=0.0159, FDR multiple comparison adjustment (mono.basal vs mono.Insulin, p= 0.4418; multi.basal vs multi.Insulin.10 p= 0.2695). **I.** Exocytosis rate constants determined from HA-GLUT4-GFP exocytosis assays in day-3 differentiated SKM-GLUT4 cells in basal (unstimulated) and with 10 nM insulin stimulation for 30 minutes. Dashed lines join individual experiments. Bar graphs showing the mean. *p* value, Student's 2 sample t-test. **J.** Endocytosis rate constants determined from HA-GLUT4-GFP endocytosis assays in day-3 differentiated SKM-GLUT4 cells, in basal (unstimulated) and with 10 nM insulin stimulation for 30 minutes. Lines join data of individual experiments. Bar graphs show the mean. ns, non-significant using Student's paired two-tailed t-test.

Journal of Cell Science • Supplementary information

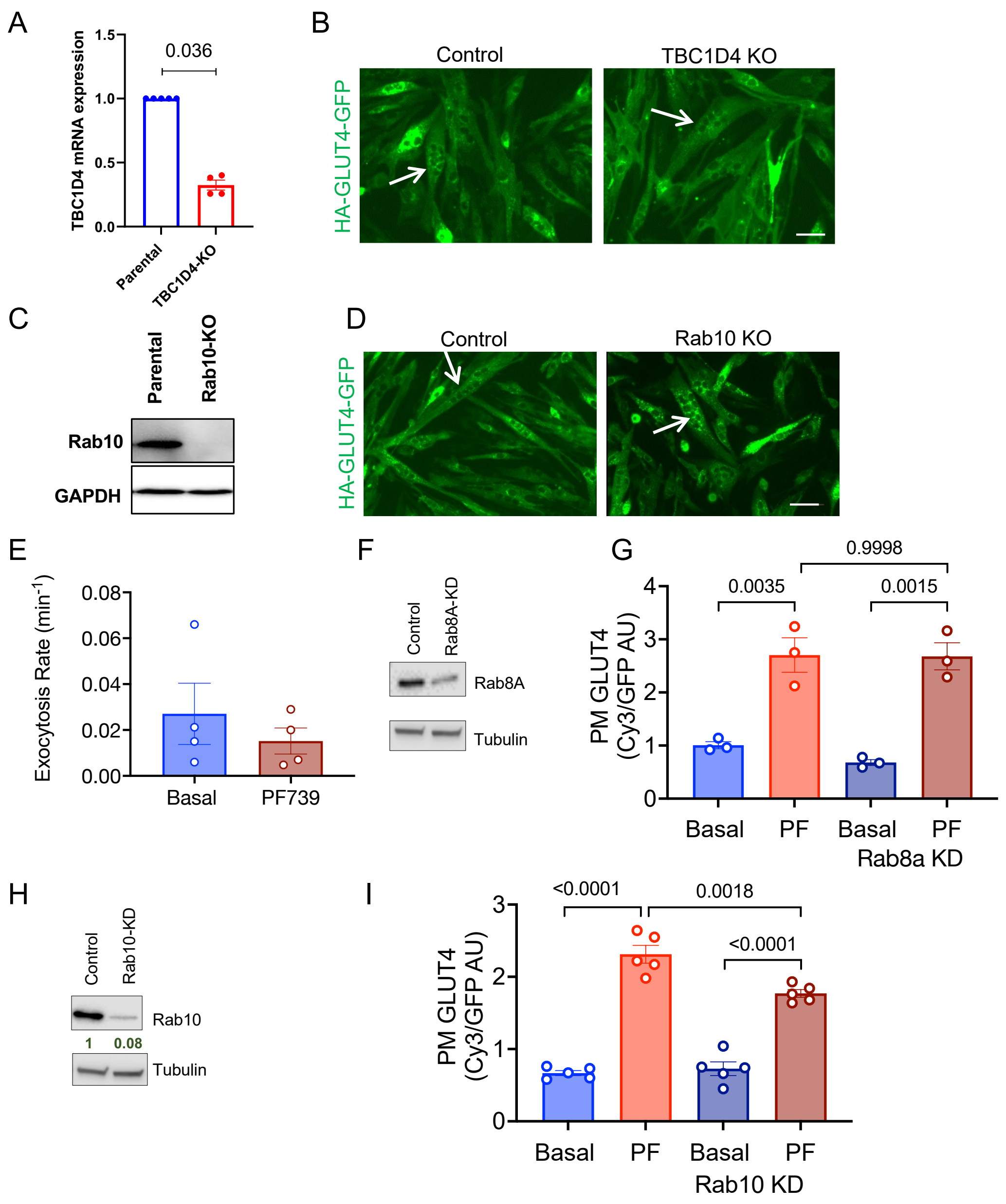

Fig. S2. in support of Figure 2.

A. TBC1D4 mRNA expression in TBC1D4 KO cells. Two-sided paired Student's T test on non-normalized data. Student's 2 sample t-test. **B.** Fluorescence imaging for HA-GLUT4-GFP in differentiated SKM-CRISPR/CAS9 (control) and TBC1D4 KO SKM cells. Arrows note multinuclear cells. Scale bar 20µm. **C.** Assessment of Rab10 knockout by western blotting. **D.** Fluorescence imaging for HA-GLUT4-GFP in differentiated SKM-CRISPR/CAS9 (control) and Rab10 KO SKM cells. Arrows indicate multinuclear cells. Scale bar 20µm. **E**. GLUT4 rate constants measured in 4 independent experiments of control and Rab10 KO cells in unstimulated and PF739-stimulated conditions. **F.** Rab8a knockdown (KD) measured by western blotting. **G.** PM GLUT4 in unstimulated and PF739 stimulated control and Rab8a KD cells. Each symbol are data from independent experiments. Two-way ANOVA with Tukey's correction for multiple testing. **H.** Rab10 KD in SKM cells measured by western blotting. **I.** PM GLUT4 in unstimulated and PF739 stimulated control and Rab10 KD cells. Each symbol are data from independent experiments. Each symbol are data from independent experiments. Two-way ANOVA with Tukey's correction for multiple testing.

Journal of Cell Science • Supplementary information

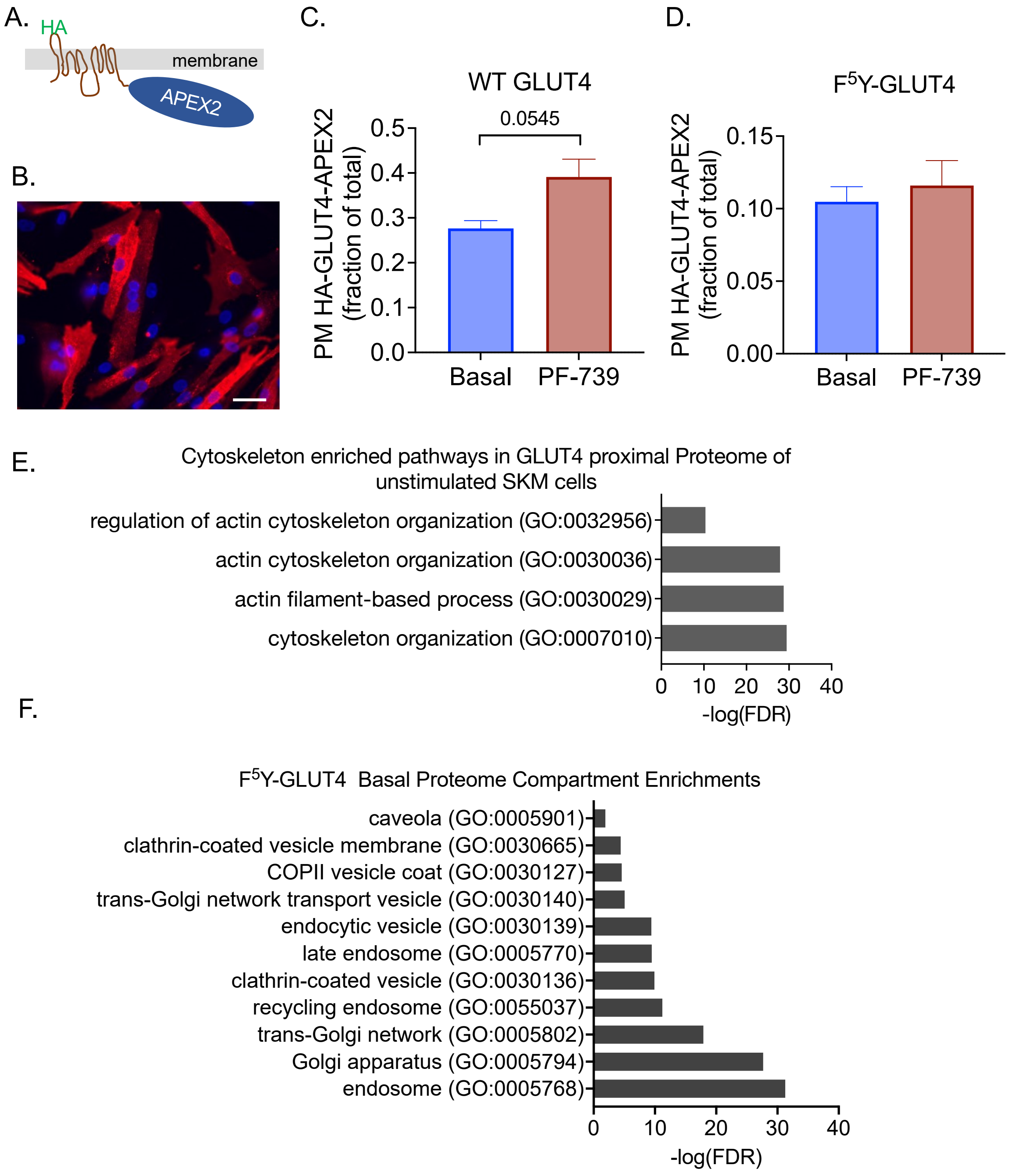

Fig. S3 in support of Figure 3.

A. Cartoon of HA-GLUT4-APEX2. APEX2 replaces GFP of HA-GLUT4-GFP.

B. Immunofluorescence of permeabilized SKM cells stably expressing HA-GLUT4-APEX2 stained with anti-HA antibody.

C. Translocation of HA-GLUT4-APEX2 to the PM of SKM cells by PF739 activation of AMPK. Unlike measurement of PM HA-GLUT4-GFP, which is a single cell ratiometric determination using GFP fluorescence power to normalize the anti-HA signal per cell, measurement of PM HA-GLUT4-APEX2 is a cell population measurement in which the average PM anti-HA staining of cells (fixed, intact cells) from one dish is divided by the total HA-GLUT4-APEX2 expression measured in a separate dish by anti-HA IF staining of fixed, permeabilized cells. This is necessary because the APEX2 construct does not have a second tag that can be used to normalize to total expression per cell. Because these are a population rather than single cell normalized PM measures, the net translocation (that is, fold increase of PF739-stimulated over unstimulated) is smaller than that determined using ratiometric measurements (that is, values corrected for expression per cell), thereby accounting for the apparently smaller translocation of HA-GLUT4-APEX2 than HA-GLUT4-GFP. Welch's 2-sample t-test. **D.** Translocation of HA-F^5Y-GLUT4-APEX2 to PM of SKM cells measured as discussed for panel C. **E.** Some cytoskeleton pathways highly enriched among proteins of the GLUT4 proximal proteome in unstimulated cells. **F.** Some pathways highly enriched among proteins of the F^5Y-GLUT4 proximal proteome in unstimulated cells. Ontology enrichment analyses were performed using Panther online software[79-81].

Table S1. WT GLUT4-APEX2 proximal proteome in unstimulated SKM cells.

Available for download at
https://journals.biologists.com/jcs/article-lookup/doi/10.1242/jcs.261454#supplementary-data

Table S2. WT GLUT4-APEX2 proximal proteome in PF739 stimulated SKM cells.

Available for download at
https://journals.biologists.com/jcs/article-lookup/doi/10.1242/jcs.261454#supplementary-data

Table S3. WT GLUT4-APEX2 proximal proteome in PF739 stimulated SKM cells contrasted to unstimulated cells.

Available for download at
https://journals.biologists.com/jcs/article-lookup/doi/10.1242/jcs.261454#supplementary-data

Table S4. F5Y-GLUT4-APEX2 proximal proteome in unstimulated SKM cells.

Available for download at
https://journals.biologists.com/jcs/article-lookup/doi/10.1242/jcs.261454#supplementary-data

Table S5. F5Y-GLUT4-APEX2 proximal proteome in PF739 stimulated SKM cells.

Available for download at
https://journals.biologists.com/jcs/article-lookup/doi/10.1242/jcs.261454#supplementary-data

Table S6. Effect of AMPK activation of F5Y-GLUT4 unstimulated proximal proteome.

Available for download at
https://journals.biologists.com/jcs/article-lookup/doi/10.1242/jcs.261454#supplementary-data

Journal of Cell Science • Supplementary information
