# Supplementary material for: Regulated dynamic subcellular GLUT4 localization revealed by proximal proteome mapping in human muscle cells
Source: J Cell Sci. 2023 Dec 21;136(23):jcs261454. doi: 10.1242/jcs.261454 (PMC10753500; doi:10.1242/jcs.261454)
Supplement: Supplementary file 1 [file joces-136-261454_review_history.pdf]

Regulated dynamic subcellular GLUT4 localization revealed by proximal proteome mapping in human muscle cells

Anuttoma Ray, Jennifer Wen, Lucie Yammine, Jeffrey Culver, Isabella Supardi Parida, Jeonifer Garren, Liang Xue, Katherine Hales, Qing Xiang, Morris J. Birnbaum, Bei B. Zhang, Mara Monetti and Timothy E. McGraw
DOI: 10.1242/jcs.261454

Editor: Jennifer Lippincott-Schwartz

Review timeline

Original submission:	5 July 2023
Editorial decision:	22 August 2023
First revision received:	29 September 2023
Accepted:	21 November 2023

Original submission

First decision letter

MS ID#: JOCES/2023/261454

MS TITLE: GLUT4 dynamic subcellular localization is controlled by AMP kinase activation as revealed by proximal proteome mapping in human muscle cells

AUTHORS: Anuttoma Ray, Jennifer Wen, Lucie Yammine, Jeffrey Culver, Jeonifer Garren, Liang Xue, Katherine Hales, Qing Xiang, Morris J Birnbaum, Bei B Zhang, Mara Monetti, and Timothy E McGraw
ARTICLE TYPE: Research Article

We have now reached a decision on the above manuscript.

To see the reviewer's reports and a copy of this decision letter, please go to: https://submit-jcs.biologists.org and click on the 'Manuscripts with Decisions' queue in the Author Area. (Corresponding author only has access to reviews.)

As you will see, the reviewer gave a favourable report but raised some critical points that will require amendments to your manuscript. I hope that you will be able to carry these out because I would like to be able to accept your paper, depending on further comments from the reviewer.

Please ensure that you clearly highlight all changes made in the revised manuscript. Please avoid using 'Tracked changes' in Word files as these are lost in PDF conversion.

I should be grateful if you would also provide a point-by-point response detailing how you have dealt with the points raised by the reviewer in the 'Response to Reviewers' box. Please attend to all of the reviewer's comments. If you do not agree with any of their criticisms or suggestions please explain clearly why this is so.

Reviewer 1

© 2023. Published by The Company of Biologists under the terms of the Creative Commons Attribution License (https://creativecommons.org/licenses/by/4.0/).

Advance summary and potential significance to field

In this study, Ray et al have analyzed AMPK-regulated GLUT4 trafficking routes in a muscle cell line using GLUT4 reporter constructs and APEX2 proximity mapping. They confirm the requirement of TBC1D4 and the Rab-GTPase Rab10 (instead of previously reported Rab8) for GLUT4 translocation in muscle cells. Cell surface labeling of GLUT4 provides evidence that the AMPK activator PF739 alters both exocytosis (increase) and endocytosis (decrease) of the transporter, leading to an accumulation of GLUT4 at the PM. The basal and AMPK-stimulated GLUT4 proximal proteomes were relatively similar, suggesting similar routes of GLUT4 traffic in basal and stimulated cells. The authors suggest that both intracellular GLUT4 retention and AMPK regulated translocation involve a dynamic sorting mechanism that is somewhat in contrast to previous studies published by the James lab.

The study provides important new knowledge in the context of GLUT4 translocation. In particular, the use of a novel human muscle cell line is significant and highlights possible differences in frequently used models. The manuscript is written well, and the conclusions are generally supported by the experimental data. However, a few questions remain and should be addressed.

Comments for the author

Main
1) the authors use a proprietary human muscle cell line that is much less characterized as the commonly used rodent lines, such as L6 and C2C12. In order to validate the model, more details about the cells should be included in the manuscript. For instance, do these cells express endogenous GLUT4? Does it increase during myotube formation and does it respond to insulin, e.g. exhibit regulated translocation? What is exact source of the cell line, and could donor variability affect the results?
2) as overexpression of GLUT4 may affect trafficking routes and kinetics, what is the level of overexpression of the GLUT4 reporter(s) compared to endogenous values?
3) In mouse models, knockout of RabGAPs has been associated with reduced levels of GLUT4 protein which has been attributed to posttranslational events, i.e. missorting and lysosomal targeting. Did TBC1D4 k/d affect GLUT4 in the human cells? Did labelling identify specific lysosomal targets for GLUT4?
4) Several figures compare two genotypes and two conditions (e.g. Fig 2D con vs kd; bas vs. stim) which usually requires 2-way ANOVA , instead of 1-way ANOVA testing. Please correct.

Minor
5) The divergence of AMPK action on the rates of endo/exocytosis in rodent L6 cells vs human cells is quite intriguing. Could you please speculate on these apparent differences?
6) How do the kinetic parameters, e.g. rate constants, t1/2 for translocation etc relate to values from the literature, such as those determined by the Holman lab using chemical surface labels? Adipocytes?
7) In muscle tissue, the effect size of insulin and AMPK activation on GLUT4 translocation/glucose uptake is roughly similar. Is this also the case in the human cell line?
8) Please correct (multiple) typos in text (e.g. WY GLUT4) and figure labels e.g. Fig 3F,..Fig5E, G, H

First revision

<u>Author response to reviewers' comments</u>

We thank the reviewer for her/his thoughtful comments on the manuscript and for providing guidance for how to improve the clarity of the presentation of our findings. We have modified the manuscript according to the reviewer's comments. All changes are noted in yellow highlighting. . Our responses to the comments are provided below.

Reviewer 1 Comments for the Author:

© 2023. Published by The Company of Biologists under the terms of the Creative Commons Attribution License (https://creativecommons.org/licenses/by/4.0/).

1) the authors use a proprietary human muscle cell line that is much less characterized as the commonly used rodent lines, such as L6 and C2C12. In order to validate the model, more details about the cells should be included in the manuscript. For instance, do these cells express endogenous GLUT4? Does it increase during myotube formation and does it respond to insulin, e.g. exhibit regulated translocation?

Although we have not characterized endogenous GLUT4 in the SKM cells, GLUT4 mRNA expression, measured by qPCR, is increased at day 3 and further at day 5 of differentiation when compared to the expression in undifferentiated SKM cells (Reviewer Figure 1). These data support differentiation dependent induction of GLUT4 expression. We have not further characterized native GLUT4 in these cells. We characterized the effect of insulin in studies of HA-GLUT4-GFP.

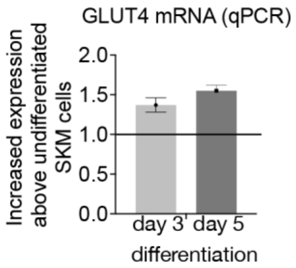

Reviewer Figure 1. Induction of native GLUT4 mRNA expression in SKM cells with differentiation. Expression (ΔΔCT) normalized to GAPDH. Average ± SD on two experiments.

What is exact source of the cell line, and could donor variability affect the results?

Thank you for this comment. SKM cells are an immortalized human muscle cell line derived from GIBCO human skeletal myoblast cells (Thermo Fisher, A11440) by stable lentiviral transduction with hTERT and CDK4 (Cokorinos et al., 2017, PMID: 28467931). This clarifying information has been added to the method section (page 16 revised manuscript). It is impossible to know whether donor variability affects the results, which is true for all clonal cell lines used in cell-bases studies.

2) as overexpression of GLUT4 may affect trafficking routes and kinetics, what is the level of overexpression of the GLUT4 reporter(s) compared to endogenous values?

Thank you for this comment. We appreciate the concern raised regarding the amount of expression HA- GLUT4-GFP impacting its behavior, an issue we have previously addressed in studies of then reporter in cultured adipocytes (PMID: 14595108 and 16269413). Accurately assessing the relative expression of HA-GLUT4-GFP to endogenous GLUT4 per cell is challenging because we do not study a clonal line but rather a pool of SKM cells expressing HA-GLUT4-GFP, therefore western blotting is not appropriate. It is critical to address the potential that the amount of HA-GLUT4-GFP expression (in all or a significant fraction of cells) saturates a step of GLUT4 trafficking and thereby bias the results.

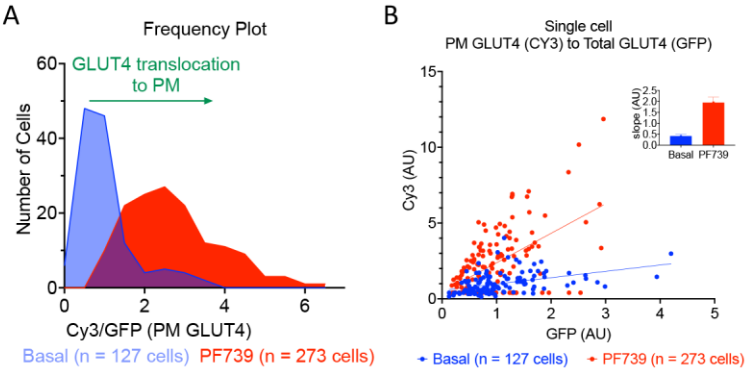

Reviewer Figure 2. Single cell analyses of HA-GLUT4- expression and sorting/trafficking of the reporter. **A.** Frequency distribution plot of Cy3/GFP per cell for basal (unstimulated) population of cells (n=127) and PF739 stimulated cells (n=273). Stimulation with 1 µM for 60 min. **B.** Plot of Cy3 and GFP fluorescence powers per cell. Inset are slopes of the Cy3 versus GFP lines for each condition. The data are pooled from 3 independent experiments.

© 2023. Published by The Company of Biologists under the terms of the Creative Commons Attribution License (https://creativecommons.org/licenses/by/4.0/).

To address potential saturation, we examine the behavior of the HA-GLUT4- GFP reporter in single cells accounting for the amount of expression of the reporter expressed per cell. To do this we use the GFP fluorescence power per cell as a measure of the HA-GLUT4-GFP expression. A frequency distribution plot of the Cy3/GFP ratio of HA-GLUT4-GFP (PM expression) per cell in basal and PF739 stimulated (1 µM 60 min) SKM cells demonstrates roughly normal distributions of data in both conditions, and that upon stimulation the population shifts to the right (increased PM HA-GLUT4-GFP)(**Reviewer Figure 2A**). A plot of Cy3 (PM) versus GFP (total) for individual cells demonstrate a proportional increase of surface GLUT4 (Cy3) with increasing total expression of HA-GLUT4-GFP (GFP, x-axis) (**Reviewer Figure 2B**). If expression achieved saturation there would be a discontinuous jump in the data as the expression amount exceeds the capacity to properly sort/traffic HA-GLUT4-GFP. This is not the case, establishing HA-GLUT4-GFP is not expressed at an amount that saturated the soring machinery.

The slopes of the lines are proportional to the fraction of HA-GLUT4-GFP on the PM. The ratio of the slopes is therefore proportional to the degree of GLUT4 increase in the PM following stimulation, which for these data is approximately 4 -fold (**Reviewer Figure 1B inset**). Based on the reviewer's comment, we have included reviewer Reviewer Figure 1B and inset as supplemental Figure 1B that is described on page 5 of the revised manuscript.

3) In mouse models, knockout of RabGAPs has been associated with reduced levels of GLUT4 protein which has been attributed to posttranslational events, i.e. missuiting and lysosomal targeting. Did TBC1D4 k/d affect GLUT4 in the human cells?

Thank you for this comment. As noted by the reviewer, previous reports have shown that knockouts of RabGAPs result in changes in GLUT4 amounts, presumably due to post translational mechanisms. However, we did not see a significance difference in steady state expression of HA-GLUT4-GFP, based on GFP fluorescence power, between parental and TBC1D4 knockout cells, although the coefficient of variance for expression of HA-GLUT4-GFP in the TBC1D4 knockout cells was greater than in control SKM cells (**Reviewer Figure 2**). There are many possible explanations for the difference in findings, perhaps most obviously differences between cultured cells and in vivo tissue. We provide these data for the reviewer but since we do not have an explanation for the differences in findings we have not modified the manuscript. To address this disparity requires detailed pulse chase studies to accurately determine the half-life of GLUT4.

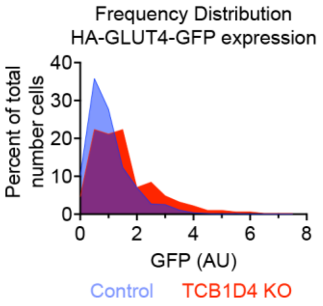

Reviewer Figure 2. Frequency distribution of HA- GLUT4-GFP expression (GFP fluorescence power) in control and TBC1D4 KO cells. 553 control cells and 497 TBC1D4 KO cells.

Did labelling identify specific lysosomal targets for GLUT4?

Protein ontology pathway analyses showed an enrichment of late endosomal proteins among the 700 proteins of the GLUT4 proximal proteome in unstimulated SKM-GFP cells as well as an enrichment in some ubiquitin ligases (Figure 3C and Supplemental table 1 of the manuscript). However, to address the reviewer's comment requires GLUT4 APEX2 mapping in TBC1D4 KO SKM cells, a future direction of our work but beyond the scope of the current manuscript.

4) Several figures compare two genotypes and two conditions (e.g. Fig 2D con vs kd; bas vs. stim) which usually requires 2-way ANOVA , instead of 1-way ANOVA testing. Please correct.

In the figures noted, the interaction term in the two-way ANOVA does not reach the threshold cutoff and thus we reduced the model and only compare main effects, which we believe is the appropriate data analyses and therefore we have not changed the figures. However, the significant results (i.e., $p < 0.05$) from the two-way ANOVA with an interaction term are the same as the one-

© 2023. Published by The Company of Biologists under the terms of the Creative Commons Attribution License (https://creativecommons.org/licenses/by/4.0/).

way ANOVA although the absolute p values are different as presented below.

Figure 2D significant differences two-way ANOVA with Tukey's correction for multiple testing:
WT Basal vs PF739 stimulated: $p < 0.0001$.
Rab10 KO, Basal vs PF739 stimulated: $p = 0.0062$.
WT PF739 vs Rab10 KO PF739 stimulated: $p = 0.0012$.

Figure Supplement 2G significant differences two-way ANOVA with Tukey's correction for multiple testing:
WT Basal vs PF739 stimulated: $p = 0.0035$.
Rab8 KO, Basal vs PF739 stimulated: $p = 0.0015$.
WT PF739 vs Rab8 KO PF739 stimulated: $p = 0.9998$.

Figure Supplement 2I significant differences two-way ANOVA with Tukey's correction for multiple testing:
WT Basal vs PF739 stimulated: $p < 0.0001$.
Rab10 KD, Basal vs PF739 stimulated: p = $p < 0.0001$.
WT PF739 vs Rab10 KD PF739 stimulated: $p = 0.0018$.

Minor

5) The divergence of AMPK action on the rates of endo/exocytosis in rodent L6 cells vs human cells is quite intriguing. Could you please speculate on these apparent differences?

Our results establish that AMPK activation induces GLUT4 translocation in human muscle cells both by inhibition of endocytosis and acceleration of exocytosis. These data highlight potential differences between human and rodent cells because AMPK activation in rodent cells has been previously shown to induce GLUT4 translocation in L6 rat muscle cells by inhibition of endocytosis only. This apparent difference between the human and rodent cell lines may be attributed to the possible differences in the molecular regulators (e.g., Rabs, Rab GAPs, etc.) targeted downstream of AMPK activation, which may in turn differentially regulate exocytosis or endocytosis. We appreciate the reviewer's comment, but we have not modified the manuscript because we can only speculate on the differences at this time.

6) How do the kinetic parameters, e.g. rate constants, t1/2 for translocation etc relate to values from the literature, such as those determined by the Holman lab using chemical surface labels? Adipocytes?

In L6 myotubes, the GLUT4 internalization rate constant in the basal conditions is reported to be very rapid: 0.43 min^{-1} or 43% or PM GLUT4 internalized per minute (Fazakerley et al. PMID:19915010). In that study insulin stimulation and AICAR activation decreased GLUT4 internalization to 0.18 min^{-1} and 0.16 min^{-1}, respectively. Those data indicate that both insulin and AMPK target GLUT4 internalization. It is worth noting that an internalization rate constant of 0.43 min^{-1} measured by Fazakerley is twice the internalization rate of the transferrin receptor and LDL receptor, and therefore is extremely rapid. Fazakerley's et al finding that insulin inhibits GLUT4 internalization is in contrast to extensive work from the Klip lab demonstrating insulin does not inhibit GLUT4 internalization in L6 (rodent) muscle cells (e.g., PMID:16418206). Thus, there are some discrepancies in how GLUT4 is regulated in L6 muscle cells.

In our study of human SKM cells, the GLUT4 internalization rate constant in the basal steady state was 0.15 min^{-1} and was further decreased during the steady-state responses to PF739 (0.05 min^{1}) (values from Figure 1I). An internalization rate constant of 0.05 min^{-1} is in line with the value for bulk membrane uptake in fibroblast cell types (e.g., PMID 9575164).

In our study, the GLUT4 exocytosis rate constant in the basal steady state was 0.03 min^{-1} in contrast to adipocytes (3T3-L1 cells), in which the basal state GLUT4 exocytosis rate constant is > 0.01 min^{-1} (PMID 14595108). The slower exocytosis of GLUT4 in adipocytes agrees with the greater intracellular retention of GLUT4 in those cells as compared to SKM cells: <5% on PM of adipocytes and ~15% on PM of SKM cells. In adipocytes insulin stimulation promotes a near 10-fold acceleration of GLUT4 exocytosis rate constant (0.08 min^{-1}) accounting for the large redistribution of GLUT4 to the PM of adipocytes. Control of GLUT4 exocytosis in SKM cells is less dramatic than in adipocytes. Upon insulin stimulation of SKM cells, GLUT4 exocytosis rate constant is accelerated to 0.07 min^{-1} and to 0.09 min^{-1} by AMPK stimulation (values from Figure 1G). Although these rates are similar to the stimulated rates in adipocytes (and also approximately the exocytosis rates of transferrin

© 2023. Published by The Company of Biologists under the terms of the Creative Commons Attribution License (https://creativecommons.org/licenses/by/4.0/).

receptor), the net translocation in SKM cells is smaller because the amount of GLUT4 in the PM of basal SKM cells is higher.

7) In muscle tissue, the effect size of insulin and AMPK activation on GLUT4 translocation/glucose uptake is roughly similar. Is this also the case in the human cell line?
No, the effect size of insulin and AMPK activation on GLUT4 translocation is different in the muscle cell line as compared to that in the muscle tissue. While AMPK results in a 2.9 ± 0.17-fold increase of PM GLUT4 in multinucleated human SKM cells, relative to basal PM GLUT4 (Figure 1E), insulin stimulation induced a 51 ± 17% (mean ± SEM) increase of PM GLUT4 relative to that in unstimulated cells (Figure 1H). Our data suggest the greater fold translocation of GLUT4 downstream of insulin receptor stimulation compared to insulin stimulation reflects both a faster exocytosis in AMPK stimulated cells (0.09 min-1) versus insulin 0.07 moin-1 and the fact that AMPK stimulation slows GLUT4 internalization whereas insulin does not. The kinetics data for AMPK regulation of GLUT4 are presented in Figure 1G-J and for insulin in Figure S1H-J.

8) Please correct (multiple) typos in text (e.g. WY GLUT4) and figure labels e.g. Fig 3F,..Fig5E, G, H
Thank you for pointing out those typos. We have made the necessary changes.

Second decision letter

MS ID#: JOCES/2023/261454

MS TITLE: GLUT4 dynamic subcellular localization is controlled by AMP kinase activation as revealed by proximal proteome mapping in human muscle cells

AUTHORS: Anuttoma Ray, Jennifer Wen, Lucie Yammine, Jeffrey Culver, Isabella Supardi Parida, Jeonifer Garren, Liang Xue, Katherine Hales, Qing Xiang, Morris J Birnbaum, Bei B Zhang, Mara Monetti, and Timothy E McGraw
ARTICLE TYPE: Research Article

I am happy to tell you that your manuscript has been accepted for publication in Journal of Cell Science, pending standard ethics checks.

Reviewer 1

Advance summary and potential significance to field

Many thanks to the authors who have adequately addressed the issues and answered the questions.

Comments for the author

There has been an issue related to the statistics (Fig. 2D,G, S2L). Technically, a two-way ANOVA is the appropriate test for a comparison of two genotypes and two conditions when only comparing the main effect, i.e. not considering the interaction. Thankfully, the authors now provide the corresponding p-values from the 2-way ANOVA in the response letter to the reviewer, and it is strongly suggested that those values are included in the manuscript, thus replacing the values from the (in this case inappropriate) 1-way ANOVA analyses.

© 2023. Published by The Company of Biologists under the terms of the Creative Commons Attribution License (https://creativecommons.org/licenses/by/4.0/).
